# Evaluating the performance of raw and epoch non-wear algorithms using multiple accelerometers and electrocardiogram recordings

**DOI:** 10.1038/s41598-020-62821-2

**Published:** 2020-04-03

**Authors:** Shaheen Syed, Bente Morseth, Laila A. Hopstock, Alexander Horsch

**Affiliations:** 1 Department of Computer Science, UiT The Arctic University of Norway, Tromsø, Norway; 2 School of Sport Sciences, Faculty of Health Sciences, UiT The Arctic University of Norway, Tromsø, Norway; 3 Department of Community Medicine, Faculty of Health Sciences, UiT The Arctic University of Norway, Tromsø, Norway

**Keywords:** Epidemiology, Medical research

## Abstract

Accurate detection of accelerometer non-wear time is crucial for calculating physical activity summary statistics. In this study, we evaluated three epoch-based non-wear algorithms (Hecht, Troiano, and Choi) and one raw-based algorithm (Hees). In addition, we performed a sensitivity analysis to provide insight into the relationship between the algorithms’ hyperparameters and classification performance, as well as to generate tuned hyperparameter values to better detect episodes of wear and non-wear time. We used machine learning to construct a gold-standard dataset by combining two accelerometers and electrocardiogram recordings. The Hecht and Troiano algorithms achieved poor classification performance, while Choi exhibited moderate performance. Meanwhile, Hees outperformed all epoch-based algorithms. The sensitivity analysis and hyperparameter tuning revealed that all algorithms were able to achieve increased classification performance by employing larger intervals and windows, while more stringently defining artificial movement. These classification gains were associated with the ability to lower the false positives (type I error) and do not necessarily indicate a more accurate detection of the total non-wear time. Moreover, our results indicate that with tuned hyperparameters, epoch-based non-wear algorithms are able to perform just as well as raw-based non-wear algorithms with respect to their ability to correctly detect true wear and non-wear episodes.

## Introduction

Accelerometers are increasingly used as an objective tool to study daily physical activity (PA)^1–4^. Currently, accelerometers are capable of measuring the body’s acceleration in all three spatial axes and are used as a proxy for PA intensity and duration^5^. Accelerometers offer versatility, minimal participation burden, relative cost efficiency^6,7^, and they also limit the information bias commonly found in PA self-report measures, such as questionnaires, activity logs and diaries^8–10^. Besides collecting data on PA intensity and duration, accelerometers have also been used successfully for activity type recognition^11,12^, body posture and movement classification^13^, energy expenditure prediction^14^, and sleep pattern estimation^15^.

Presently, there exists an overwhelming amount of accelerometry data collection and processing criteria addressing a variety of research needs^16,17^. Examples include minimum daily wear time^18^, body placement of the accelerometer^19^, the use of raw recordings (i.e., gravity units) compared with count-based recordings^20^, cut-points for intensity classification^21^, and determination of accelerometer non-wear time^16^. However, the latter, determination of the time during which the accelerometer is not worn (non-wear time), has received little attention in the literature^22^, and numerous studies have failed to address it entirely^16,23^. Determining the non-wear time of the accelerometer is important in assessing study compliance and accurately calculating of summary statistics^5^, such as minutes spent sedentary, or in light, moderate or vigorous activity^24,25^.

Several non-wear detection algorithms, which typically look for periods of zero acceleration within specified time intervals (epochs), have been developed^26–28^. In addition to epoch-based non-wear algorithms, algorithms that work on raw acceleration data have also been developed^29,30^. Such methods typically examine the standard deviation and value ranges of the acceleration axes within a certain time interval and associate low values with non-wear time. One of the major challenges of non-wear algorithms is that episodes of true non-wear time are frequently indistinguishable from episodes of sleep or sedentary time. In particular, epoch-based non-wear methods, in which raw accelerometry data (e.g., sampled at 100 Hz) is collapsed into a single value for a specified time interval (e.g., 60 sec), have been shown to frequently misclassify non-wear time as wear time and vice versa^31^.

A few studies have examined the accuracy and validity of existing non-wear algorithms and their influence on PA summary statistics. Most recently, researchers have studied the accuracy of non-wear algorithms by comparing their results to logbook data^22,32–34^. Although these comparisons are illuminating, the limitations inherent in self-report measures^35^ remain a source of error when considering the participant’s logbook as the gold standard. Additionally, logbooks may not yield the precision needed to determine when a device was worn and not worn^25^. Other validation studies have explored the built-in proximity sensors of accelerometers and used skin contact data to determine true wear and non-wear time episodes^36^. However, since the proximity sensor requires close contact with the skin, vibration movement or additional layers (e.g., hip bands or clothing) may have caused the reported 17.8% misclassification of wear-time as non-wear time in the study cited above, making such methods sub-optimal.

This paper builds on previous validation studies to examine the performance of existing non-wear algorithms, both for algorithms that use epoch data and those that use raw acceleration data recorded in gravity units (*g*). In particular, we focused our attention on four commonly used non-wear algorithms frequently employed in PA studies^26–30^. We created a gold standard dataset by combining free-living acceleration data collected by two triaxial accelerometers worn simultaneously, one of which also provided a full single-channel electrocardiogram (ECG) waveform. We trained a machine learning model that was able to classify episodes of true non-wear time with up to one-minute precision, from large discrepancies between the signal data of the two accelerometers. Additionally, we conducted a sensitivity analysis to study the relationship between input and output variables for each of the four algorithms. In other words, we examined the effects of varying the hyperparameter values on the algorithms’ ability to accurately detect non-wear time. Our objective here was three-fold: (i) to evaluate the performance of the four non-wear algorithms on a gold standard dataset; (ii) to provide insight into the relationship between an algorithm’s classification performance and its hyperparameter values; and (iii) to provide optimized hyperparameter values that will assist in more accurately detecting non-wear episodes from free-living accelerometry data.

## Background

This section provides details on the four non-wear algorithms explored in this study, namely, the Hecht^27^, Troiano^28^, Choi^26^, and Hees^29,30^ algorithms. For the sake of simplicity, we often use the surname of the first author to refer to each of the algorithms. We describe how each of the algorithms works, and what hyperparameters and default values are involved. Hecht, Troiano, and Choi are epoch-based non-wear algorithms that work on the number of counts in specified intervals (epochs), whereas Hees works on raw acceleration data. It is important to note that a count is a dimensionless unit and is neither inherently meaningful nor interpretable^37^. Counts are proprietary units and typically derived from an unknown algorithm as part of accelerometer software, such as ActiLife (ActiGraph, LLC, Pensacola, FL, USA). A count also significantly reduces the information present in the underlying raw data. For example, 10 seconds of raw data sampled at 100 Hz would yield 100 $\times$ 10 = 1,000 data points per axis. In contrast, counts within a 10 second epoch would be collapsed into a single value per axis.

### Hecht algorithm

In a paper by Hecht *et al*.^27^, 22 subjects with chronic obstructive pulmonary disease (COPD) participated in a study aimed at developing a novel method for analyzing accelerometer data that produces an accurate picture of the daily activity of the participants. A key part of the method was an algorithm used to detect wear and non-wear time on a minute-by-minute basis. Participants wore an RT3 triaxial accelerometer (Stayhealthy, Monrovia, CA) on the non-dominant hip for a two week period, and the accelerometer recorded activity counts in 1 minute epochs. During the measurement period, participants were not given any instructions as to their daily activity patterns. The algorithm used to detect wear and non-wear time was based on the following three questions for each 1 minute epoch: (i) Is the VMU CMP (vector magnitude in counts per minute) value > 5.0; (ii) of the following 20 minutes, do at least 2 minutes have VMU CPM values > 5.0; and (iii) of the preceding 20 minutes, do at least 2 minutes have VMU CPM values > 5.0 (to allow for spikes or artificial movement). If at least two of the conditions were affirmed, then the 1 minute epoch was considered to be wear time; otherwise, it was considered to be non-wear time. The hyperparameters and corresponding default values of their algorithm included:

- The VMU CMP threshold; defaults to 5.0.
- The following ("upstream”) and preceding ("downstream”) activity window size; defaults to 20 minutes.
- The number of "spikes” in the following or preceding activity windows; defaults to 2.

### Troiano algorithm

In a study by Troiano *et al*.^28^, PA levels of 11,196 children, adolescents, and adults were studied with an accelerometer that recorded counts in 1 minute epochs. Participants were asked to wear an ActiGraph model 7164 uniaxial accelerometer (ActiGraph, LLC; Ft. Walton Beach, FL) on their right hip for a seven day period. They were instructed to wear the accelerometer during all waking hours, except for during swimming or bathing. After the data collection phase, the authors developed an algorithm to classify time intervals into wear and non-wear episodes. The non-wear episodes were defined as intervals of at least 60 consecutive minutes of zero activity counts, with allowance for 1–2 minutes of counts between 0 and 100 (to allow for spikes or artificial movement). All remaining data were considered to be wear time. The hyperparameters and corresponding default values of their algorithm included:

- The minimum length of the interval to classify an episode as non-wear time; defaults to 60 minutes.
- The number of spikes in the interval; defaults to 2.
- The lower bound of the spikes threshold within the interval; defaults to 0 counts.
- The upper bound of the spikes threshold within the interval; defaults to 100 counts.

The Troiano algorithm was developed for a uniaxial accelerometer; however, the algorithm can easily be extended to operate on triaxial accelerometer data by calculating the VMU of the three axes. To do so, a binary hyperparameter, "use VMU", which defaults to "no", was added.

### Choi algorithm

A study by Choi *et al*.^26^ was designed to validate the accuracy of the Troiano algorithm^28^. A total of 49 adults and 76 adolescents wore an ActiGraph GT1M accelerometer (ActiGraph, Pensacola, FL, USA) inside a whole-room indirect calorimeter on their dominant hip for a 24 hour period. The ActiGraph GT1M recorded counts at 1 second epochs, which were then summed to 60 second epochs. The authors compared the Troiano algorithm with the accelerometer wearing status measured by the whole-room indirect calorimeter. Their analysis showed that the Troiano algorithm tended to misclassify episodes of sedentary behavior as non-wear time (type I error). Thus, the authors devised a new algorithm as a proposed improvement on the Troiano algorithm. The improved algorithm classified episodes of at least 90 minutes with consecutive zero counts as non-wear time, while allowing for a short time interval with non-zero counts lasting up to 2 minutes, if no counts were detected during a 30 minute upstream or downstream window from that interval. The algorithm consisted of the following hyperparameters and associated default values:

- The minimum length of the interval to classify an episode as non-wear time; defaults to 90 minutes.
- The number of spikes in the interval; defaults to 2.
- The upstream or downstream window size; defaults to 30 minutes.
- The number of spikes in the upstream or downstream window; defaults to 0.

Similar to the Troiano algorithm, the Choi algorithm was originally developed for a uniaxial accelerometer but can be extended to work on triaxial accelerometers by calculating the VMU. To do so, a binary hyperparameter, “use VMU”, which defaults to “no”, was added.

### Hees algorithm

A study by van Hees *et al*.^29^ examined whether or not a simple summary measure derived from raw triaxial accelerometer data was able to contribute to the estimation of PA-related energy expenditure in pregnant and non-pregnant women. A total of 108 women from Sweden and 99 women from the United Kingdom wore a triaxial GENEA accelerometer (GENEA, Unilever Discover, Sharnbrook Bedfordshire, UK) for 10 and 7 day periods, respectively. To detect periods of wear and non-wear time, the authors developed an algorithm in which episodes of wear and non-wear time were estimated based on the standard deviation and the value range of each of the three accelerometer axes in 30 minute intervals. The 30 minute intervals were considered non-wear time if the standard deviation was less than 3.0 m*g* (1 m*g* = 0.00981 m$\cdot$s${}^{-2}$) for at least 2 out of 3 axes, or if the value range was less than 50 m*g* for at least 2 of the 3 axes. The algorithm consisted of the following hyperparameters and associated default values:

- The minimum length of the interval to classify an episode as non-wear time; defaults to 30 minutes.
- The standard deviation threshold; defaults to 3.0 m*g*.
- The minimum number of axes for the standard deviation threshold; defaults to 2.
- The value range threshold; defaults to 50 m*g*.
- The minimum number of axes for the value range threshold; defaults to 2.

In another study by van Hees *et al*.^30^, the length of the interval was increased to 60 minutes to decrease the chance of incorrectly classifying sedentary episodes as non-wear time (type I error). Additionally, a sliding window of 15 minutes was used to allow for overlapping episodes and to more precisely detect the boundaries of the non-wear episodes.

A summary of the available hyperparameters and default values for each of the four non-wear algorithms is presented in Table 1.

**Table 1:** Overview of hyperparameters and default values as part of the Hecht, Troiano, Choi, and Hees non-wear algorithms.

Hyperparameter description	Hecht	Troiano	Choi	Hees
VMU threshold	5 VMU			
Upstream and downstream window size (mins)	20 mins		30 mins	
Number of spikes (artificial movement) in window	1		0	
Minimum interval (mins)		60 mins	90 mins	30 mins
Number of spikes (artificial movement)		2	2	
Minimum spike threshold (counts)		0		
Maximum spike threshold (counts)		100		
Calculate the VMU of 3 axes (yes/no)		no	no	
Standard deviation threshold (m*g*)				3 m*g*
Minimum number of axes to check the std. threshold				2
Value range threshold (m*g*)				50 m*g*
Minimum number of axes to check the value threshold				2

## Methods

### Participants and study protocol

The Tromsø Study is a population-based cohort study in the municipality of Tromsø, Norway, and include seven data collection waves taking place between 1974 and 2016^38^. Data used in this study were derived from the seventh wave of the Tromsø Study (Tromsø 7) conducted from 2015–2016^39^. Tromsø 7 was approved by the Regional Committee for Medical Research Ethics (REC North ref. 2014/940) and the Norwegian Data Protection Authority, and all participants gave written informed consent. The usage of data in this study has been approved by the Data Publication Committee of the Tromsø Study.

All inhabitants of the municipality aged 40 years and older (n = 32,591) were invited to Tromsø 7, of which 21,083 (65%) attended. Among these, a random sample (n = 6,778) were invited to wear an ActiGraph wGT3X-BT accelerometer (ActiGraph, LLC, Pensacola, United States), of which 6,333 accepted. Among these 6,333 participants, 698 participants were randomly selected to simultaneously wear a second accelerometer, the Actiwave Cardio (CamNtech Ltd, Cambridge, UK), for at least 24 hours.

The participants attended two separate examinations during April 2015 and October 2016. The first examination included clinical examinations, biological sampling, and questionnaires. Height and weight were measured with standard methods by trained personnel, and participants were instructed to wear light clothing without shoes during the measurements. Body mass index (BMI) was calculated as weight divided by height squared (kg/m${}^{2}$). The second examination was scheduled a few weeks after the first examination and included more extensive clinical examinations. During the second examination, all participants were provided with ActiGraph and Actiwave Cardio accelerometers. After data had been collected, the devices were returned by mail.

Because the Actiwave Cardio additionally records a single-channel ECG waveform (besides recording acceleration), obtaining valid data was highly dependent on the correct placement of the ECG pads onto the participants’ chests. Due to the sensitivity of the sensors, recordings from 22 participants contained measurement or device errors (e.g., one axis producing out of range acceleration values, or no recording at all) and were removed from further analyses. In addition, 93 participants had missing metadata or stopped participating partway through the study. Valid data from both devices was finally gathered concerning 583 participants, 267 (45.8%) males and 316 (54.2%) females, aged 40-84 (mean = 62.74; SD = 10.25). The participants had a mean height of 169.81 cm (SD = 9.35), a mean weight of 78.31 kg (SD = 15.27) and a mean body mass index of 27.06 kg/m${}^{2}$ (SD = 4.25).

### Accelerometers

The participants each wore an ActiGraph model wGT3X-BT accelerometer (ActiGraph, Pensacola, FL) with a dynamic range of $\pm$ 8 *g* (1*g* = 9.81 m$\cdot$s${}^{-2}$) that recorded raw acceleration in gravity units (*g*) along three axes (vertical, mediolateral and anteroposterior). The ActiGraph accelerometer was fully charged and initialization was performed using ActiLife software (ActiGraph, LLC, Pensacola, United States). The sampling frequency was set to 100 Hz, and the accelerometer was placed on the right hip, on an adjustable elastic waistband, for seven consecutive days and nights. The participants were instructed to wear the ActiGraph accelerometer at all times, except during water-based activities and contact sports.

At the same time, the participants were wearing an Actiwave Cardio accelerometer (CamNtech Ltd, Cambridge, UK) with a dynamic range of $\pm$ 8 *g* that recorded raw acceleration data along three axes as well as a full single-channel ECG waveform. The Actiwave Cardio was fully charged and initialized by entering each participant’s sex, weight and height, as well as start time and date into the Actiwave Cardio system software (CamNtech Ltd, Cambridge, UK). This process also included setting up the ECG recordings (128 Hz sample rate; 9-bit resolution) and accelerometer recordings (32 Hz sample rate). The Actiwave Cardio was then attached to each participant’s chest using ECG pads. Before attaching the ECG pads, body hair at the position of the pads was removed and skin cleaned, if needed. The Actiwave Cardio was then calibrated by a 40 m walking and 2 minute resting test. Since the Actiwave Cardio is light-weight and waterproof, the participants were instructed to wear it at all times, including during water-based activities, such as showering and swimming.

During the data collection phase, the GT3X files from the ActiGraph accelerometers and the EDF (European Data Format) files from the Actiwave accelerometers were downloaded and stored for offline analyses. For the sake of readability, we will from now on use the terms ActiGraph and Actiwave to refer the ActiGraph and Actiwave Cardio devices.

### Data pre-processing

The GT3X file is a zip-compressed file containing meta- and acceleration data in a binary format. Python scripts were developed to directly read the content of both files so as to automate the data processing workflow without relying on graphical user interface software. The EDF file, which contained the triaxial acceleration data, the single-channel ECG wavelet, and the heart rate in beats per seconds (derived once per second from the ECG data) was read using the Python library *pyedf*. All data were subsequently stored in Hierarchical Data Format 5 (HDF5) for further analysis.

The ActiGraph and Actiwave data were synchronized based on the recorded timestamps with the nanosecond-scale precision required to synchronize 100 Hz and 32 Hz data. The ActiGraph contained data from seven full days, whereas the Actiwave contained data for roughly 24 hours, after which the battery had drained. During the device initialization phase, the Actiwave began recording immediately after it was attached to the participant. The ActiGraph, however, was set to begin recording at midnight (00:00) the following day.

After joining the ActiGraph and Actiwave data, an average of 17.4 hours (SD = 4.5) of data per participant remained. The joining of the data from the two devices was performed by inner joining on the data timestamps after downscaling the ActiGraph data from 100 Hz to 32 Hz using the band-limited Whittaker-Shannon (i.e., Sinc) interpolation method for sampling rate conversion^40^. In addition, the Euclidian norm minus one (ENMO) was calculated for both streams of raw triaxial acceleration data, as $\max \{\sqrt{ac{c}_{x}^{2}+ac{c}_{y}^{2}+ac{c}_{z}^{2}}-1,0\}$, where $ac{c}_{x}$, $ac{c}_{y}$, and $ac{c}_{z}$ refer to each of the orthogonal axes, respectively^30,41^, and negative values were rounded to zero. ActiGraph counts per 10 second epoch were obtained using ActiLife’s (version 6.13.4; no filters) built-in raw-to-count conversion algorithm and were then summed to counts per 60 second epoch, these being suitable for the Hecht, Troiano and Choi non-wear algorithms.

### Finding candidate non-wear episodes

A candidate non-wear episode was defined as an episode or interval of no activity that shows characteristics of true non-wear time but cannot yet be classified as true non-wear time. For example, candidate non-wear episodes may occur during sleeping, sedentary behavior as well as during true non-wear time; which were of interest to our analysis. Candidate non-wear episodes were detected by calculating the standard deviation of the ActiGraph raw triaxial data for each 1 minute interval. By visual inspection of the data, a standard deviation threshold of $\le$4.0 m*g* (0.004 *g*), recognizable by horizontal or flat plot lines, was appropriate to obtain candidate non-wear episodes. More concretely, lowering this threshold would not detect any episode of no activity, meaning that 4.0 m*g* is very close to the accelerometer’s noise level. Consecutive 1 minute intervals were grouped into candidate non-wear episodes and their start and end time were stored.

**Figure 1:**
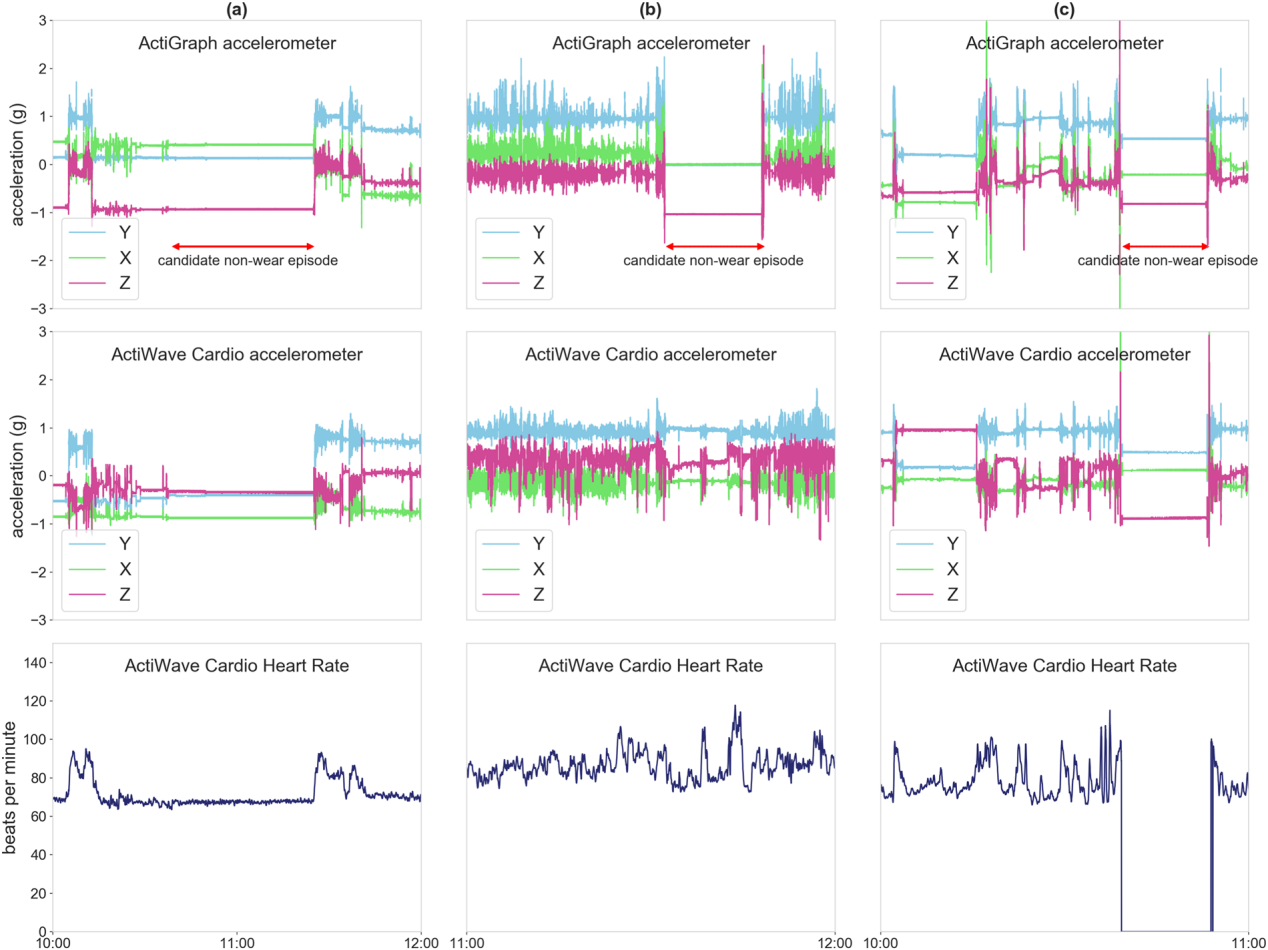
Three scenarios in which candidate non-wear time episodes were detected: (**a**) both accelerometers were worn (not true non-wear time); (**b**) the ActiGraph was not worn (true non-wear time), and (**c**) both accelerometers were not worn (true non-wear time).

To distinguish episodes of true non-wear time from episodes of sedentary time or sleep, we used the acceleration and heart rate recordings from the simultaneously worn Actiwave. In other words, we used the data recorded by the Actiwave to help determine what in particular was happening during candidate non-wear episodes. The detected candidate episodes could have occurred in three different scenarios: (i) both accelerometers were worn (not true non-wear time); (ii) the ActiGraph was not worn (true non-wear time), and (iii) both accelerometers were not worn (true non-wear time). By focusing on discrepancies between the data from both accelerometers, we were able to distinguish between sedentary and sleep episodes, and episodes of true non-wear time. A visual depiction of the three scenarios, labeled (a), (b), and (c), respectively, is shown in Fig. 1. Further details on the three scenarios are as follows:

1. The ActiGraph and the Actiwave recorded no activity (flat lines). There existed little discrepancy between the acceleration signals, while a valid heart rate was detected by the ECG recording. Here, both devices were worn, and the candidate non-wear episode cannot be classified as true non-wear time (Fig. 1(a)).
2. The ActiGraph recorded no activity, while the Actiwave recorded some activity, including a valid heart rate from the ECG signal. If the ActiGraph was worn, it should have produced an acceleration signal following a similar activity pattern to that of the Actiwave acceleration signal. Here, the discrepancy between the two devices is large and the corresponding acceleration signals are “out-of-sync”, and the candidate non-wear episode was classified as true non-wear time. This scenario is shown in Fig. 1(b).
3. The ActiGraph and the Actiwave recorded no activity and there was no heart rate in the ECG signal. Here, both devices were not worn, and the candidate non-wear episode can be classified as true non-wear time. This scenario is shown in Fig. 1(c) and occurred very infrequently in our dataset.

Since our objective here was to find true non-wear episodes from the ActiGraph device alone, scenarios involving candidate non-wear episodes from the Actiwave device were not further considered.

### Training a machine learning classifier

The candidate non-wear episodes from a random subset from 250 of the 583 participants (912 episodes) were manually labeled as true non-wear time or wear time by visual inspection of the acceleration data in accordance with the three scenarios described in the previous section. The features of each candidate non-wear episode were derived from the ActiGraph ENMO, the Actiwave ENMO, and the Actiwave heart rate data. For each of the three types of data, the following features were calculated: mean, standard deviation, min, max, kurtosis, signal-to-noise ratio (i.e., mean divided by the standard deviation), mode, median, range, variance, and the lower bound of the dynamic time warp (DTW)^42^. The differences between features from the two accelerometer recordings were additionally included. For example, we included the difference between the variance from the ActiGraph ENMO and Actiwave ENMO. This was done for all features except the lower bound of the DTW. For each candidate non-wear segment, a total of 41 features were constructed.

All features were standardized by removing the mean and then scaled to unit variance. To allow for some non-linearity in classifying episodes, second-degree polynomial features were created for all features and combinations of features. We explored the following four machine learning algorithms and one deep learning algorithm to build a classification model: (1) support vector machine (SVM)^43^ with linear, polynomial, sigmoid, and radial basis function kernels; (2) logistic regression; (3) decision trees; (4) adaptive boosting^44^, and (5) the multi-layer perceptron. All candidate episodes from the 250 participants were shuffled and stratified into 80% training and 20% test data. Training was done with 10-fold cross-validation and the machine learning hyperparameters of the four classifiers were tuned using a random grid search approach (Supplementary Table S1). All models were created using the Python library, Scikit-learn^45^ on a workstation with a dual Xeon 20-core/40-thread CPU and 512 GB of RAM running Linux. Total wall clock time for training with 10-fold cross validation and exploring roughly 42,000 different hyperparameters was six days.

The classification performance of the five classifiers on the training and test set is shown in the Supplementary Table S2. The hyperparameters that resulted in the best classifiers are shown in the Supplementary Table S3. The SVM machine learning classifier performed best on the test set, with a near-perfect F1 score of 0.996, misclassifying only 4 of 912 non-wear episodes as wear time. A visual inspection of the misclassified episodes showed that candidate non-wear episodes of long durations (>2 hours) were misclassified as wear time (false negatives; type II error). The SVM classifier was then applied to the remaining 583–250 = 333 participants to classify the candidate non-wear episodes into true non-wear time and wear time. All episodes were visually inspected, and all misclassified long-duration episodes were programmatically corrected. In doing so, we created a ground truth (i.e., gold standard) dataset which included true non-wear time episodes of 583 participants on a minute-by-minute basis. This dataset was subsequently used to study the classification performance of the Hecht, Troiano, Choi and Hees algorithms.

### Implementing the four non-wear algorithms

The Hecht, Troiano, Choi, and Hees algorithms were implemented in the Python programming language according to their published descriptions and, where available, their published online code^46^. The algorithms’ hyperparameters were implemented as arguments that could take on any value, rather than being fixed to their default values. This allowed for hyperparameter tuning and conducting a sensitivity analysis. Our Troiano and Choi implementations were validated by implementation in the ActiLife software package, and Choi was additionally validated against its original implementation in the SAS programming language^46^. Our Hees implementation was validated against its own GGIR implementation^47^ in the R programming language. Our Hecht implementation could not be validated against an external tool or open-source implementation. Instead, two authors independently implemented the Hecht algorithm in Python and Matlab by following the decision tree described in the original publication^27^. The implementations were then validated against each other and no differences were found.

### Calculating classification performance

**Figure 2:**
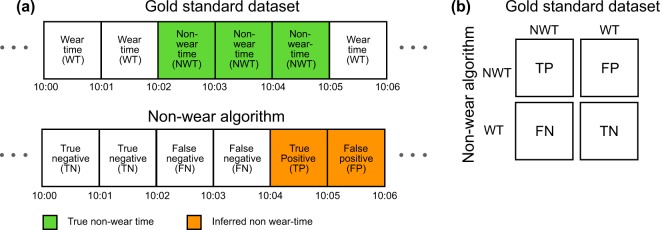
Graphical representation showing the correct and incorrect classifications of wear and non-wear time: **(a)** six minutes of labeled data is compared against six minutes of inferred data derived from a non-wear algorithm. Depending on the difference between the true labels and the inferred labels, each 1-minute interval is classified as a true negative or true positive if the labels match. Alternatively, intervals are classified as false negatives or false positives of the labels mismatch. **(b)** similarly as **(a)** but now shown as a confusion matrix.

The gold standard dataset contained data measured on a minute-by-minute basis whether the device was worn (wear time) or not (non-wear time). We executed the Hecht, Troiano and Choi algorithms on the 60 second epoch data, and the Hees algorithm on the raw triaxial ActiGraph data. All implementations were executed using the algorithms’ default hyperparameter values, as outlined in Table 1. The resulting non-wear vectors were compared to the gold standard dataset on a minute-by-minute basis, as shown in Fig. 2.

True non-wear time inferred as non-wear time contributed to the true positives (TP), and true wear time inferred as wear time contributed to the true negatives (TN). Both TPs and TNs are necessary to obtain a high accuracy of the non-wear time algorithms, as they are the correctly inferred classifications. True non-wear time inferred as wear time contributed to the false negatives (FN), and true wear time inferred as non-wear time contributed to the false positives (FP). Both FNs and FPs will result in an overall lower accuracy, which is calculated by $\frac{TP+TN}{TP+TN+FP+FN}$. Besides accuracy, we calculated three other classification performance metrics. Precision was calculated as $\frac{TP}{TP+FP}$, recall as $\frac{TP}{TP+FN}$, and F1 as the harmonic mean of precision and recall, $2\times \frac{precision\times recall}{precision+recall}$. Recall represents the fraction of correctly inferred non-wear time in relation to all the true non-wear time—in medicine, this is also known as sensitivity. In the example depicted in Fig. 2, the recall would be 0.33, as we correctly inferred only 1 minute out of a total of 3 minutes of true non-wear time. Precision shows the fraction of correctly inferred non-wear time in relation to all inferred non-wear time. In the example in Fig. 2, the precision would be 0.5, since we correctly inferred only 1 minute out of a total of 2 minutes non-wear time. Typically, both precision and recall are important, and the F1 score captures their harmonic mean as a single value. A perfect recall can easily be obtained by inferring every minute as non-wear time; however, most of the inferred non-wear time would then be incorrect, resulting in a very low precision. Alternatively, a perfect precision can be obtained by only correctly classifying a single minute as non-wear time and the remainder as wear time; however, we would then only detect a very small percentage of the total non-wear time in the data which results in a very low recall. In this paper, we cared equally about recall and precision, and throughout the remainder of the paper we mainly report results that are optimized for F1.

### Sensitivity analysis and hyperparameter tuning

To study the effects of input variables (i.e., hyperparameter values) on output variables (i.e., classification performance), we performed a sensitivity analysis by executing the four non-wear algorithms with a variety of hyperparameter values. In total, 18,203 different hyperparameter value combinations were explored (Supplementary Table S4). The chosen values were structured as ranges and were typically fractions or multiples of the algorithms’ default values. For example, if an interval length had a default value of 60 minutes, we explored the values 1, 30, 60, 90, and 120. While empirically investigating the effect of varying the hyperparameters on the performance of the algorithms (accuracy, precision, recall and F1), we also obtained optimized hyperparameter values by mean of hyperparameter tuning on all the data. In addition, we conducted hyperparameter tuning with 10-fold cross-validation by randomly splitting and stratifying the data into 70% training and 30% test data. In doing so, we aimed to obtain optimized hyperparameters by reducing overfitting to the training data, thus improving generalizability.

## Results

### Overview of non-wear time episodes

**Figure 3:**
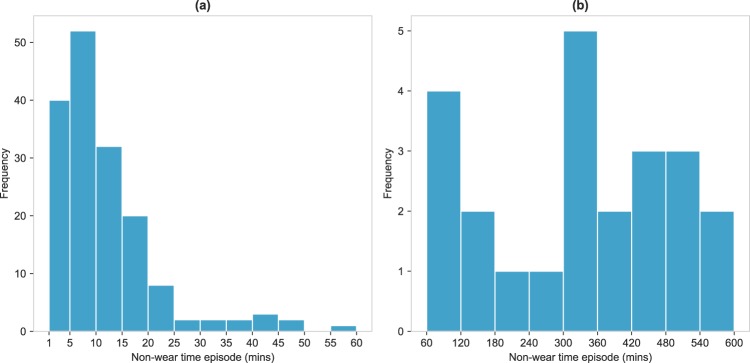
Distribution of non-wear time episodes. **(a)** <60 minutes, (**b**) $\ge$60 minutes.

In total, 188 episodes of non-wear time were present in our gold standard dataset. The majority of these were non-wear episodes lasting <60 minutes, which accounted for 164 (87.2%) episodes and had a mean duration of 11.0 minute (SD = 9.90). Non-wear episodes lasting $\ge$60 minutes accounted for 24 (12.8%) of the episodes and had a mean duration of 355 minutes (SD = 204.1), or roughly 6 hours. Fig. 3 shows the frequency distribution of episodes lasting <60 minutes (a) and $\ge$60 minutes (b). The distribution of episode durations <60 minutes was highly skewed, with most of these episodes lasting between 1–15 minutes. Episodes $\ge$60 minutes had a more uniform distribution, with episodes between 5–6 hours occurring the most frequently. Supplementary Fig. S1 provides an overview of what time of day episodes of non-wear time occurred, revealing that most of the non-wear time occurred during the morning hours, from 06:00–07:00 (11.8%) and 07:00–08:00 (9.9%). During these hours, approximately 75% of the episodes lasted <60 minutes. Non-wear episodes lasting $\ge$60 minutes occurred most frequently during the night phase, from 00:00–08:00. In total, non-wear time contributed to 172 hours (1.7%) of a total of 10,123 hours of data.

### Classification performance of the four non-wear time algorithms

**Figure 4:**
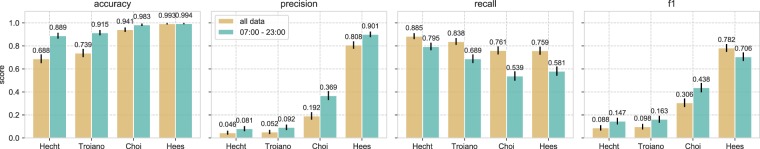
Classification performance of the four non-wear algorithms with their default hyperparameter values on all the data, and a subset of the data (07:00–23:00). Error bars indicate the 95% confidence interval.

 Figure 4 shows the classification performance of the four non-wear algorithms with their default hyperparameter values. The original Troiano algorithm was not optimized to detect non-wear episodes during the night phase, as the accelerometer was removed during sleep in the original study^26,28^. Besides showing the results obtained from our total data, Fig. 4 additionally presents the classification performance from a filtered subset of our gold standard dataset; in which we zoom in on data occurring only during the day phase (07:00–23:00).

The percentage of correctly inferred non-wear time in relation to all the inferred non-wear time was captured by the precision score. The Hees algorithm—which works on raw acceleration data—was able to obtain the highest precision score by classifying up to 90% of all the inferred non-wear time correctly. Among the epoch-based algorithms, Hecht (4.6%) and Troiano (5.2%) both performed poorly on all the data, as well as on the day phase data (8.1% and 9.2%, respectively). The Choi algorithm obtained a moderate precision score during the day phase (36.9%) and performed poorly (19.2%) when taking all the data into account.

The percentage of correctly inferred non-wear time in relation to all the true non-wear time was captured by the recall score. In contrast to precision, all algorithms scored higher on their recall performance. When considering all of the data, Hecht detected 88.4%, Troiano 83.8%, Choi 76.1%, and Hees 75.9% of the total true non-wear time. The recall values during the day phase were lower for all algorithms, indicating that detecting true-non wear time episodes during the day is more difficult using the algorithms’ default hyperparameters.

The F1 metric captures both precision and recall equally, providing a single value metric to capture how much non-wear time is detected (recall), while simultaneously looking at how much of the inferred non-wear time is correct (precision). Hecht and Troiano performed poorly on the F1 metric, both in the total data as well as that of the day phase. While their recall score was high, the low precision scores indicate the presence of many false positives (the misclassification of true wear time as non-wear time). In other words, Hecht and Troiano overestimated the amount of non-wear time in the data. Choi performed moderately on the F1 metric, especially during the day phase (0.44), while it did obtain the best F1 score among the three epoch-based algorithms. The Hees algorithm outperformed all epoch-based algorithms on the F1 metric, with scores of 0.78 and 0.70 for the total data and the day phase, respectively.

### Sensitivity analysis

In this section, we report on the effects that the hyperparameter input values had on the algorithm’s classification performance (i.e., output variable) and provide insight into how hyperparameter values that deviate from the default values are able to contribute to increased classification performance. The presented contour plots display a three-dimensional view of varying two hyperparameter values and the effect on the algorithm’s F1 performance (which captures both precision and recall). Note that when the number of hyperparameters exceeded two, the remaining hyperparameters were fixed to the default value (see Table 1), and the presented results should be interpreted accordingly (i.e., *ceteris paribus*). Contour lines that remained parallel to the x-axis when varying the hyperparameter values of the x-axis had little effect on classification performance, as their value remained constant. Likewise, contour lines that remained parallel to the y-axis when varying the value on the y-axis had little effect on classification performance.

#### Hecht

**Figure 5:**
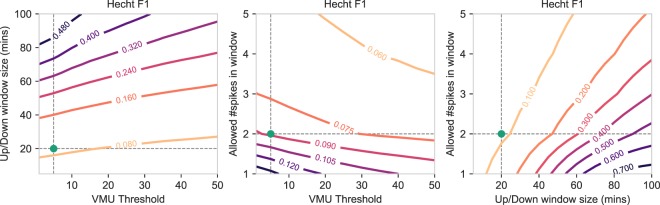
Contour plots showing how the F1 score of the Hecht non-wear algorithm changes when varying the hyperparameter values shown on the x-axis and y-axis, while keeping the remaining hyperparameters fixed to the default values. Additionally, the green dot indicates the default value of the x-axis and y-axis as originally published in the Hecht study.

The Hecht algorithm (Fig. 5) showed a large variation in F1 scores when varying the size of the window, with an increased window size leading to an increased F1 performance. Similarly, the number of spikes within the window showed much variation, with a lower number of spikes contributing to a higher F1 score. The VMU threshold showed little variation, although increasing the VMU threshold resulted in a slight decrease in F1 performance when simultaneously increasing the window size or increasing the number of spikes in the window. The Hecht algorithm was able to achieve a higher F1 score with a larger window size, while allowing less artificial movement (i.e., spikes) to occur in that window. This also had a positive effect on the precision performance (Supplementary Fig. S3), where a larger window combined with a lower number of spikes was associated with higher precision scores. However, increasing the window size had a negative effect on the recall performance (Supplementary Fig. S4). In summary, our results showed that using a larger window in combination with allowing less artificial movement within that window resulted in a higher F1 performance with more correctly inferred non-wear time (higher precision). However, an increased window was negatively associated with the total true non-wear time detected (lower recall).

#### Troiano

**Figure 6:**
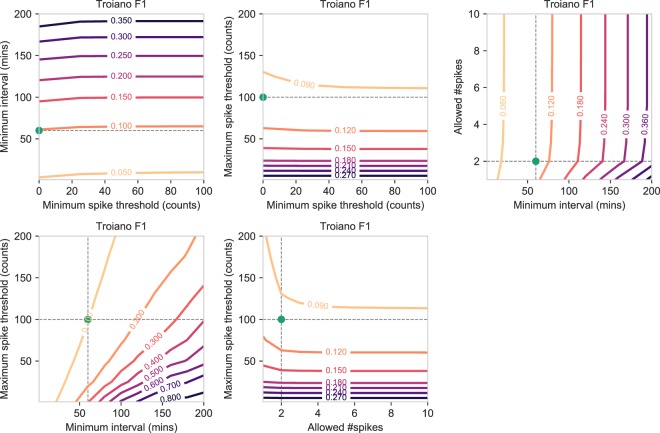
Contour plots showing how the F1 score of the Troiano non-wear algorithm changes when varying the hyperparameter values shown on the x-axis and y-axis, while keeping the remaining hyperparameters fixed to the default values. Additionally, the green dot indicates the default value of the x-axis and y-axis as originally published in the Troiano study.

For the Troiano algorithm (Fig. 6), a larger time interval—the minimum length of a non-wear episode—was associated with an increased F1 performance. The number of spikes allowed in an interval had little impact, however, the upper bound of the spike value (maximum spike threshold) showing increased F1 performance with lower values. Additionally, the lower bound of the spike threshold—also the value that constitutes a zero count—was not associated with an increase in F1 performance in any case. The performance of the Troiano algorithm was also significantly improved by increasing the length of the interval while decreasing the upper bound of the spike threshold. A lower spike threshold was associated with less intense artificial movement, although the frequency of artificial movement had little effect on F1 performance. The precision performance also showed an increase when increasing the length of the interval while decreasing the maximum spike threshold (Supplementary Fig. S6). However, an increased interval had a negative effect on recall performance (Supplementary Fig. S7). In summary, our results showed that increasing the window size while decreasing the upper bound of the spike threshold resulted in an overall higher F1 performance and more correctly inferred non-wear time (higher precision), although the total amount of true non-wear time detected was lower (lower recall).

#### Choi

**Figure 7:**
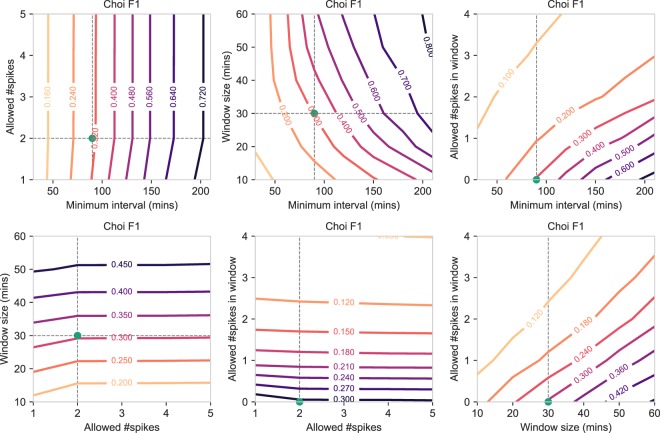
Contour plots showing how the F1 score of the Choi non-wear algorithm changes when varying the hyperparameter values shown on the x-axis and y-axis, while keeping the remaining hyperparameters fixed to the default values. Additionally, the green dot indicates the default value of the x-axis and y-axis as originally published in the Choi study.

The Choi algorithm combines both an interval and a window when detecting non-wear time. For the Choi algorithm (Fig. 7), the results showed similar characteristics to those of Hecht and Troiano. A larger interval was able to significantly improve F1 performance, as was an increased window length. The frequency of spikes in the interval caused little variation in F1 performance, whereas the frequency of spikes in the window was associated with a decrease in F1 performance for higher values. Similar patterns were found for precision (Supplementary Fig. S9), where larger intervals and windows were associated with increased precision performance. In contrast, a larger interval and a larger window decreased the overall recall performance (Supplementary Fig. S10), although this decrease was not as large as the decreases found when using the Hecht and Troiano algorithms. In summary, increasing the length of the interval and the length of the window were both associated with an increased F1 performance, with more correctly inferred non-wear time (higher precision). The number of spikes in the interval had little effect on overall performance, whereas lowering the number of spikes in the window increased both precision and F1. In contrast, the aforementioned effects all resulted in a slightly lower recall, with less detected true non-wear time overall.

#### Hees

**Figure 8:**
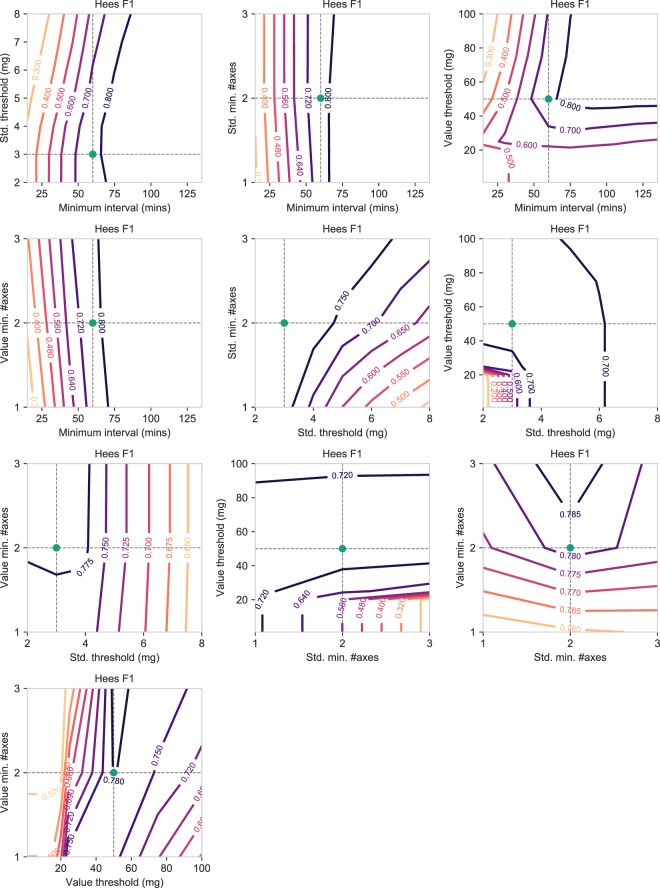
Contour plots showing how the F1 score of the Hees non-wear algorithm changes when varying the hyperparameter values shown on the x-axis and y-axis, while keeping the remaining hyperparameters fixed to the default values. Additionally, the green dot indicates the default value of the x-axis and y-axis as originally published in the Hees study.

For the Hees algorithm (Fig. 8), a longer interval was generally associated with an increased F1 performance, though it began to dwindle at a certain length. In addition, with a large interval, reducing the value threshold had a negative effect on F1 performance, whereas the standard deviation threshold had no effect on F1. Isolating the standard deviation threshold and the number of axes it was calculated from, we observed an increased classification performance when they were defined more stringently, this leading to a lower threshold calculated over more axes. Isolating the value range threshold and the number of axes it was calculated from, a mixed picture was revealed, with a sharply decreasing F1 performance for very low value ranges and the detection of non-wear time episodes becoming problematic. Similar patterns could be discerned for the accuracy (Supplementary Fig. S11) and precision scores (Supplementary Fig. S12). In concordance with Hecht, Troiano, and Choi, precision and F1 performance gains were observed to negatively impact the recall performance, although this decrease in recall was marginal in many places (Supplementary Fig. S13).

### Hyperparameter tuning

**Figure 9:**
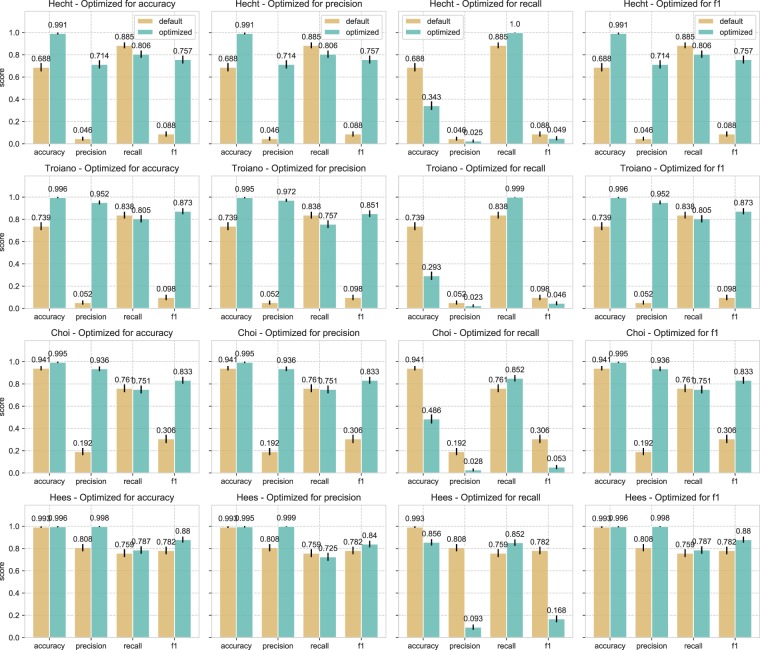
Classification performance as a result of hyperparameter tuning for accuracy, precision, recall, and F1 for Hecht, Troiano, Choi, and Hees. Each figure also shows the effect that optimizing for one metric has on the remaining metrics. For example, optimizing for recall results in a very low precision score for all four algorithms. Besides optimized scores, we also show the default classification scores obtained by using the default hyperparameter values for easy comparison; which can also be viewed in Fig. 4. Error bars indicate the 95% confidence interval.

This section presents the results of hyperparameter tuning aimed at obtaining hyperparameter values that provide the best classification performance and outlines how the obtained values deviate from the default values proposed in the algorithms’ original papers. Table 2 provides an overview of the parameters that were optimized for accuracy, precision, recall and F1. Optimizing for F1 was a tradeoff between precision and recall and the optimized values reflect consideration of both correctly inferring non-wear time and being able to detect as much true non-wear time as possible. Cross validated F1-optimized values are additionally presented; these were obtained from tuning with 10-fold cross validation to prevent overfitting to our dataset and make the obtained values more generalizable to other datasets (Supplementary Table S5). The effects of optimizing for one performance metric, and its effect on the other performance metrics, are presented in Fig. 9.

**Table 2:** Overview of default and tuned hyperparameters for accuracy, precision, recall, F1, and cross-validated (cv) F1 for Hecht, Troiano, Choi, and Hees.

Hyperparameter	Default	Accuracy	Precision	Recall	F1	F1 (cv)
**Hecht**						
VMU Threshold	5	1	1	50	1	1
Up/Down window size (mins)	20	100	100	5	100	100
Allowed #spikes in window	2	1	1	5	1	1
**Troiano**						
Minimum spike threshold (counts)	0	0–100	0–100	50–100	0–100	0
Allowed #spikes	2	1–10	1–10	1–10	1–10	1
Maximum spike threshold (counts)	100	1	1	50–200	1	1
Use VMU	no	yes	yes	yes	yes	yes
Minimum interval (mins)	60	140	200	1	140	140
**Choi**						
Minimum spike threshold (counts)	0	0–100	0–100	100	0–100	0
Allowed #spikes	2	1–5	1–5	5	1–5	1
Use VMU	no	yes	yes	no	yes	yes
Up/Down window size (mins)	30	20–60	20–60	10	20–60	20
Allowed #spikes in window	0	0–1	0–1	4	0–1	0
Minimum interval (mins)	90	210	210	30	210	210
**Hees**						
Minimum interval (mins)	60	135	90	15	135	135
Std. threshold (mg)	3	8	2	7–8	8	7
Std. min. #axes	2	2	1	1	2	1
Value threshold (mg)	50	1–100	50	1–100	1–100	1
Value min. #axes	2	1–3	2	1–3	1–3	1

#### Hecht

We achieved the highest F1 performance, 0.757 (default = 0.088), by increasing the length of the window to 100 minutes (default 20 minutes), while lowering the VMU threshold to 1 (default = 5), and setting the number of spikes in the window to 1 (default = 2). These settings also resulted in the best precision score, 0.714 (default = 0.046), while slightly decreasing the overall recall score to 0.806 (default = 0.885).

#### Troiano

Troiano performed better on all performance metrics when calculating the VMU rather than using a single axis. The best F1 performance, 0.873 (default = 0.098), was achieved by increasing the interval length to 140 minutes (default = 60) and setting the maximum spike threshold to 1 count (default = 100). The minimum spike threshold and the allowed number of spikes varied greatly, from 0–100 and 1–10, respectively, while the optimal F1 score was still maintained. The best precision score, 0.972 (default = 0.052), was achieved by increasing the window length further to 200 minutes. Optimizing for F1 and precision resulted in the lower than standard recall scores of 0.805 and 0.757, respectively (default = 0.838).

#### Choi

Similar to Troiano, Choi achieved a better accuracy, precision and F1 performance when calculating the VMU rather than using a single axis. The best F1 score of 0.833 (default = 0.306) was obtained by increasing the interval to 210 minutes (default = 90). The hyperparameters related to the allowed number of spikes in the interval and window length could also be varied from 1-5 spikes, and 20-60 minutes, respectively, while still resulting in the best F1 score (this included the default value of 2 spikes and the default 30 minute window). Tuning with cross validation, however, favored decreasing the number of spikes allowed in the interval to 1 (default = 2) and the length of the window to 20 minutes (default = 30 minutes). The tuned hyperparameter values for F1 were similar to those tuned for precision, with an optimized precision score of 0.936 (default = 0.192). In contrast to Hecht and Troiano, a recall score of 0.751, close to the default value of 0.761, was maintained when optimizing for precision and F1.

#### Hees

For Hees, an increased F1 performance of 0.88 (default = 0.782) was achieved by increasing the length of the interval to 135 minutes (default = 60). The Hees algorithm evaluates activity within an interval by calculating either the standard deviation or the value range. The tuning process favored activity detection through the value range of 1–100 m*g* and from 1–3 axes—compared to detection through the standard deviation. The best precision score of 0.999 was obtained with an interval length of 90 minutes, and slightly reducing the standard deviation threshold to 2 m*g* (default = 3 m*g*) calculated from a single axis (default = 2). Compared to the epoch-based algorithms, hyperparameter tuning for the Hees algorithm gave the smallest difference in the obtained performance scores when compared with the original values, with the largest performance gain visible in the precision metric. This is an indication that the original published hyperparameter values already work adequately on our dataset.

## Discussion

In this paper, we studied the classification performance of four commonly used epoch and raw non-wear algorithms for accelerometry data on a gold-standard dataset constructed from data on 583 participants. We also provided insight into the relationship between the algorithms’ hyperparameters and classification performance and generated optimized hyperparameter values to more accurately detect episodes of non-wear time and reduce misclassifications.

The majority of non-wear time episodes (87.2%) were shorter than 60 minutes, with the most frequent episodes lasting no longer than 10 minutes and occurring in the morning hours (06:00–08:00). Other studies have reported similar findings for 24-hour data^32^, non-sleep data^48^, as well as multi-day data^22^. Although our dataset contained no additional information (e.g., logbooks or notes), we suspect that showering in the morning was the main cause of these short non-wear episodes, and this supposition is supported by other research^22^. Non-wear algorithms that employ a large interval or window fail to detect such short episodes and this can result in a recall performance loss, i.e., increased false negatives or type II error. Because showering is not considered sedentary time, nor is it classified as PA with a high intensity level (e.g., moderate or vigorous), failing to remove such episodes would probably not have any meaningful influence on the summary statistics^22,32^. Non-wear episodes $\ge$60 minutes occurred less frequently in the total data and most frequently during the night phase. These episodes of longer duration can safely be assumed to be due to participants not wearing their sensors during sleep. Besides non-wear episodes due to sleep and showering, researchers should be careful not to unquestionably remove episodes of non-wear time if the aim is to study high intensity PA. For example, swimming or contact sports—or any other type of sports—during which the accelerometer is burdensome to wear, can significantly contribute to time spent in higher intensities. Removing these episodes under the guise of irrelevant non-wear time can cause bias in calculated summary statistics and can thus portray an incorrect picture of a person’s overall PA.

Hecht and Troiano were not designed to detect non-wear time during sleep and performed poorly on our dataset with their original default hyperparameter values. In particular, they overestimated the amount of non-wear time in the data, which increases recall, but significantly lowers precision. When applied to the filtered dataset (07:00–23:00), Hecht, Troiano and Choi performed better on the overall F1 score, albeit still worse than Hees. Note that Hees is the only examined algorithm that works on raw acceleration data rather than epoch data and significantly benefits from the detailed 100 Hz data, outperforming all the other non-wear algorithms. However, compared to the epoch-based algorithms, Hees performed slightly worse on the filtered dataset. This can be attributed to the step size of the sliding window, which defaults to 15 minutes^30^, and as a result cannot accurately detect the edges of the non-wear episodes during the day phase. Apart from computational time, we see no reason to decrease the sliding window even further (e.g., to 1 minute), which could increase recall and overall F1 performance. In contrast to previous research^49^, our results indicate that using VMU data leads to improved classification performance when compared with using data from a single axis. The use of VMU also mitigates the orientation issue caused by incorrect wearing of the accelerometer present when using a single axis^20^. Among the epoch-based non-wear algorithms, Choi performed better than Hecht and Troiano, both in precision and F1. As Choi was designed as an improvement on Troiano, as per our results, in did in fact perform better, and this is also in concordance with other research^33^. However, with tuned hyperparameters, Troiano was able to perform slightly better (F1 = 0.873) than Choi (F1 = 0.833), and similar to Hees (F1 = 0.88), which works on raw acceleration data. It is generally known that collapsing raw data into activity counts or metrics causes some loss of the information contained in the raw signal, and moreover, that this extra information could assist in improving the accuracy of predictive models^20^. However, our empirical evaluation indicates that epoch-based non-wear algorithms are able to perform just as well as raw-based non-wear algorithms in terms of their overall ability to accurately detect true non-wear and true wear episodes.

For all of the non-wear algorithms, our sensitivity analysis and hyperparameter tuning showed that there was an increase in precision and F1 performance when larger intervals or windows were used, as well as when artificial movement was defined more stringently. However, larger intervals or windows also reduced the total non-wear time detected, meaning that true non-wear time was detected as wear time, which resulted in a lower recall score (i.e., increased false negatives or type II error). These larger intervals and windows differ significantly from their original published default values. Our results indicate that Hecht performs best with a window size of 100 minutes (default 20 minutes); Troiano with an interval of 140 minutes (default 60 minutes); Choi with an interval of 210 minutes and a window of 20 minutes (default 90 and 30 minutes respectively); and Hees with an interval of 135 minutes (default 30^29^ or 60 minutes^30^). Note that though these were the intervals and windows that achieved the highest overall F1 performance, slightly shorter ones still outperformed their original counterparts. Our results are in concordance with other research; for example, Oliver *et al*.^50^ studied accelerometer data from adults recorded over a 48 hour period (excluding sleep) and reported that an interval of 180 minutes of consecutive zero counts performed best for detecting episodes of non-wear time. In another study, Jaeschke *et al*.^32^ reported that a 120 minute interval of consecutive zero counts worked best on 24 hours of continuous accelerometer data from older adults, including subsets of the data that contained waking and sleeping hours only. Hutto *et al*.^48^ also reported that a 120 minute, including a 150 and 180 minute interval of consecutive zero counts, worked best on accelerometer data of participants 50 years and older. In a study by Mailey *et al*.^51^, a 60 minute interval worked better than the shorter intervals of 20 and 30 minutes among older adults, although longer intervals were not tested. The mentioned studies had comparable age ranges to our dataset, which was 40-84 (mean = 62.74; SD = 10.25) in our dataset, indicating that the reported results should be commensurable to our findings. Compared to younger people, older people are able to wear accelerometers for longer durations without accumulating any counts^48^; as a consequence, even longer intervals or windows are necessary to prevent such long durations from being misclassified as non-wear time. In contrast to those using older participants, studies using children, adolescents, or young adults report contradictory findings. For example, Aadland *et al*.^22^ reported that a 45 or 60 minute interval of consecutive zero acceleration worked best to detect episodes of true non-wear time for accelerometer data from children 10 years and older. Van Helst *et al*.^34^ examined accelerometer data from children and adolescents over 4 and 7 day periods and also report that a shorter, 30 minute interval of zero acceleration worked best. Knaier *et al*.^33^ used accelerometer data from young adults (mean age = 25.2; SD = 4.9) and reported that a 60 minute, or even a 30 minute interval of zero counts worked best to detect episodes of non-wear time. The authors also reported that reducing the interval time from 90 minutes to 60 or 30 minutes increased the number of misclassifications of wear time as non-wear time (false positives; type I error), but that such misclassifications occurred mainly during the sleep phase. If sleep is not the primary aim, and thus excluded, a smaller window may be beneficial because it decreases true non-wear time that is misclassified as wear time (false negatives; type II error).

It is worth looking at how the default intervals and windows for the four non-wear algorithms evaluated in this study were originally established. In the original Hecht paper^27^, the authors used a window size of 20 minutes in reference to a previous study. In that study, the authors argue that the slightest movement will be recorded as a non-zero count, and a sustained 20 minute period of zero counts was judged as non-wear time^52^. Note that the 20 minute threshold was proposed in a study with middle school girls, whereas the Hecht algorithm was developed with COPD patients that had an average age of 66.8 and remained mostly sedentary^53^. A 20 minute window performed poorly on our dataset, as it resulted in a high degree of false positives (lower precision or increase type I error) and an overestimation of non-wear time. In the original Troiano paper^28^, the authors propose a 60 minute interval; however, they fail to report on the rationale behind this choice of length. Given the age range of their dataset, 6–60 years, this could be a trade-off to capture the non-wear episodes of both kids and older adults. The Choi algorithm was devised as an improvement of the Troiano algorithm, and the authors found that their algorithm worked better with a 90 minute interval and an additional 30 minute window. Additionally, the authors report a sharp decrease in false positives (higher precision; less type I error) with a 90 minute interval compared with a 60 minute interval. However, the authors recommend not increasing the interval length any further so as to not increase the number of false negatives, which would result in a lower recall or type II error. Hees originally reported an interval of 30 minutes^29^, which was increased in a later study to 60 minutes to prevent episodes of wear time being accidentally classified as non-wear time, increasing precision and lowering the number of false positives (type I error).

Altogether, the results presented in our study reveal similar patterns with regard to the trade-off between precision and recall, where longer intervals and windows generally increase precision (fewer false positives or less type I error) at the cost of decreasing recall (more false negatives or type II error). Conversely, shorter intervals and windows increase recall at the cost of decreasing precision. This trade-off is captured by the F1 score, and according to our results, longer intervals or windows drastically increase precision, while less drastically decreasing recall, resulting in an overall higher F1 score. This was especially true for Choi and Hees, for which there was only a marginal decrease in recall performance. Thus, compared to the algorithms’ default performance, most classification gains are associated with the ability to lower the false positives, or type I error, which increases precision, but does not necessarily lead to a better detection of all the true non-wear time. As a direct consequence, misclassifying wear time as non-wear time can result in an underestimation of sedentary time, and subsequently, can result in an overestimation of time spent other intensity levels^26^. This is especially problematic when the amount of sedentary time makes up the majority of acceleration data^21,54^, for instance when studying the PA of older adults^51,55^. At the same time, misclassifications can result in bias when calculating PA energy expenditure as well as in an incorrect understanding of PA, energy expenditure and health outcomes^26^.

### Strengths

One important strength of our study was our construction of a gold standard dataset (n = 583) with a minute-by-minute resolution from free-living activity. The use of a second accelerometer that included electrocardiogram recordings and the subsequently derived heart rate was also a novel approach to detecting episodes of true non-wear time without the use of additional data, such as logbooks or diaries, that might suffer from information bias in its estimation of non-wear episodes^8–10^. Our study also presented a detailed empirical analysis of the relationships between input variables and classification performance output variables (precision, recall and F1). At the same time, we evaluated the trade-off between a variety of hyperparameter values and provided insight into the relationship between false positives (type I error) and false negatives (type II error). We also provided tuned hyperparameter values for better detection of non-wear time episodes and reduction of misclassifications.

### Limitations and future research

Classification performance scores, such as precision and recall, as well as alternative metrics, such as specificity or sensitivity (which is analogous to recall), were originally developed for sets of independent points. In other words, they treat the classification of points (in this study, 1 minute activity intervals) independent of the preceding or following points, or any other points. In practice, this means that correctly classifying a single 60 minute episode of non-wear time will contribute to a better performance score than, for example, correctly classifying 59 1 minute episodes of non-wear time and incorrectly classifying another 1 minute interval. Given that all 1 minute intervals are treated independently, the latter will contain a single misclassification, whereas the former contains no misclassifications. In reality, non-wear durations are range-based, meaning that they occur over consecutive sequences of time points, and alternative measures to calculate precision and recall might be worth exploring, especially those that take into account range-specific issues^56^.

Although we evaluated the classification performance of the four non-wear algorithms on all our data, including day only (07:00–23:00) data, the sensitivity analysis and hyperparameter tuning were conducted only on all the data. The results presented in these sections might therefore only be applicable to studies using 24 hours of accelerometry data. However, there are a large number of previous studies focusing on day only data (i.e., excluding sleep). For example, in four large cross-sectional studies from England, Portugal, Norway and Sweden^3^ participants were required not to wear the accelerometer during sleep, as has been the case in other studies^57^. While our results indicate that most of the non-wear episodes <60 minutes occur during the day, and most of those $\ge$60 minutes during the night, our proposed larger intervals and windows might not be directly applicable to day only accelerometer data. Furthermore, we obtained an average of 17 hours (SD = 4.5 hours) of combined ActiGraph and Actiwave Cardio data from each participant, and collecting data over a longer time period, possibly spanning multiple days, would be worth exploring.

Our primary dataset contained accelerometry data obtained from a hip-worn accelerometer, the ActiGraph wGT3X-BT, which is the most commonly used accelerometer for PA studies^16,33^. One benefit of using hip-worn accelerometers is that it means that our evaluation is more commensurable with the original non-wear algorithm studies, albeit that we used a different type of accelerometer. Our results might not be directly applicable, however, to studies using wrist-worn accelerometers^2^. We expect movement associated with wrist-worn accelerometers to be much more intense and volatile, or have higher movement variability^20^, even during sedentary behavior. Longer sequences of zero acceleration would thus be difficult to obtain, since hand or arm movement would occur more frequently than hip movement during a sedentary episode, such as sitting. As a consequence, the distinction between sedentary episodes and true non-wear time can be more apparent with wrist-worn accelerometers, which arguably makes the detection of true non-wear time easier when using shorter intervals or windows.

## Supplementary information

Supplementary Information.

## Data Availability

The legal restriction on data availability are set by the Tromsø Study Data and Publication Committee in order to control for data sharing, including publication of datasets with the potential of reverse identification of de-identified sensitive participant information. The data can however be made available from the Tromsø Study upon application to the Tromsø Study Data and Publication Committee. Contact information: The Tromsø Study, Department of Community Medicine, Faculty of Health Sciences, UiT The Arctic University of Norway; e-mail: tromsous@uit.no. All Python code that supports this study is openly available on S.S.’s GitHub page at https://github.com/shaheen-syed/ActiGraph-ActiWave-Analysis.
