# Supplementary material for: Evaluating the performance of raw and epoch non-wear algorithms using multiple accelerometers and electrocardiogram recordings
Source: Sci Rep. 2020 Apr 3;10:5866. doi: 10.1038/s41598-020-62821-2 (PMC7125135; doi:10.1038/s41598-020-62821-2)
Supplement: Supplementary file 1 — Supplementary Information. [file 41598_2020_62821_MOESM1_ESM.pdf]

Supplementary Information

Evaluating the performance of raw and epoch non-wear algorithms using multiple accelerometers and electrocardiogram recordings

Shaheen Syed[1,*], Bente Morseth[2], Laila A. Hopstock[3], and Alexander Horsch[1]

[1]Department of Computer Science, UiT The Arctic University of Norway, Tromsø, Norway
[2]School of Sport Sciences, Faculty of Health Sciences, UiT The Arctic University of Norway, Tromsø, Norway
[3]Department of Community Medicine, Faculty of Health Sciences, UiT the Arctic University of Norway, Tromsø, Norway
[*]corresponding author: shaheen.syed@uit.no

March 21, 2020

Table S1: Overview of explored hyperparameter values when performing a random grid search during model training. Multiplier score between parentheses indicates the frequency of random values created. For example, 0.001 – 5.000 (100x) means that there are 100 random values explored between 0.001 and 5.000. Description for each of the hyperparameters can be found in the Scikit-learn documentation at `https://scikit-learn.org/`. Other hyperparameter values not listed here were kept to there default value as can additionally be found in the Scikit-learn documentation. SVM = support vector machine, ADA = adaptive, MLP = multi-layer perceptron

**Hyper-parameter**	**SVM**	**Logistic Regression**	**Decision Trees**	**ADA Boost**	**MLP**
C	0.001 – 5.000 (100x)	0.001 – 10.000 (500x)			
Kernel	linear, poly, rbf, sigmoid				
Gamma	0.001 – 2.000 (50x)				
Shrinking	True, False				
Dual		True, False			
Fit intercept		True, False			
Criterion			gini, entropy		
Splitter			best, random		
Max-features			auto, sqrt, log2, none		
N-estimator				1 – 1000 (100x)	
Algorithm				SAMME, SAMME.R	
Hidden layers					1–4
Neurons each layer					10–100 (steps of 10)
Cross-validation	10-fold	10-fold	10-fold	10-fold	10-fold
Classifiers created	400,000	20,000	160	2,000	400

Table S2: Classification performance metrics for training set (80% data with 10-fold cross-validation) and test set (20% data). Value in bold indicates the classifier that obtained the highest F1 score on the test set, and is used to classify candidate non-wear episodes into true non-wear time or wear time. ± indicate the 95% confidence interval

	Training Set	Test Set		
**Algorithm**	**F1**	**Precision**	**Recall**	**F1**
SVM	0.993	0.996 ± 0.0043	0.996 ± 0.0043	**0.996** ± 0.0043
Logistic Regression	0.994	0.988 ± 0.0072	0.988 ± 0.0071	0.987 ± 0.0072
Decision Trees	0.990	0.989 ± 0.0069	0.989 ± 0.0068	0.989 ± 0.0069
ADA Boos	0.994	0.992 ± 0.0056	0.992 ± 0.0057	0.992 ± 0.0058
Multi-layer Perceptron	0.996	0.991 ± 0.0061	0.991 ± 0.0061	0.991 ± 0.0062

Table S3: Hyperparameter values that provided the highest F1 score (see Table S2 for obtained F1 scores) for each of the four used classifiers (SVM, logistic regression, decision trees, and ADA boost). Description for each of the hyper-parameters can be found in the Scikit-learn documentation at `https://scikit-learn.org/`

Hyper-parameter	SVM	Logistic Regression	Decision Trees	ADA Boost	MLP
C	0.098	9.767			
Kernel	linear				
Gamma	0.480				
Shrinking	TRUE				
Dual		TRUE			
Fit intercept		TRUE			
Criterion			entropy		
Splitter			best		
Max-features			log2		
N-estimator				627	
Algorithm				SAMME.R	
Hidden Layers					4
Neurons each layer					70

Table S4: Overview of explored hyper-parameter values for the four non-wear algorithms investigated when performing a sensitivity analysis. The total number of combinations is shown in parentheses.

**Non-wear algorithm**	**Hyper-parameter description**	**Explored values**
Hecht (1100)	-The VMU threshold	1, 5, 10, 15, 20, 25, 30, 35, 40, 45, 50
	-The upstream or downstream window	5, 10, 15, 20, 25, 30, 35, 40, 45, 50, 55, 60, 65, 70, 75, 80, 85, 90, 95, 100
	-Number of spikes (artificial movement) allowed in window	1, 2, 3, 4, 5
Troiano (9900)	-Minimum length of an episode	1, 20, 40, 60, 80, 100, 120, 140, 160, 180, 200
	-Number of spikes allowed	1, 2, 3, 4, 5, 6, 7, 8, 9, 10
	-Lower bound of a spike value	0, 25, 50, 75, 100
	-Upper bound of a spike value	1, 25, 50, 75, 100, 125, 150, 175, 200
	-Calculate the VMU of 3 axes or use uni-axial	True, False
Choi (2100)	-The upstream or downstream window	10, 20, 30, 40, 50, 60
	-Number of spikes (artificial movement) allowed in window	0, 1, 2, 3, 4
	-Minimum length of an episode	30, 60, 90, 120, 150, 180, 210
	-Number of spikes allowed	1, 2, 3, 4, 5
	-Calculate the VMU of 3 axes or use uni-axial	True, False
Hees (5103)	-Minimum length of an episode	15, 30, 45, 60, 75, 90, 105, 120, 135
	-Standard deviation threshold	2, 3, 4, 5, 6, 7, 8
	-Minimum number of axes to check the std. threshold	1, 2, 3
	-The value range threshold	1, 5, 10, 15, 20, 25, 50, 75, 100
	-Minimum number of axes to check the value threshold	1, 2, 3

Table S5: Classification performance as a result of hyperparameter tuning with 10-fold cross validation (cv) on training data (70%) and test data (30%). Additionally, and as a comparison to using cross validation, the 'all data' column shows the classification result of hyperparameter tuning on all the data (100%). Note that when using all data, thus 100%, we did not perform cross-validation. ± indicate the 95% confidence interval

	**Hecht**			**Troiano**			**Choi**			**Hees**		
	**cv training (70%)**	**cv test (30%)**	**all data (100%)**	**cv training (70%)**	**cv test (30%)**	**all data (100%)**	**cv training (70%)**	**cv test (30%)**	**all data (100%)**	**cv training (70%)**	**cv test (30%)**	**all data (100%)**
**accuracy**	0.991 ±0.009	0.991 ±0.014	0.991 ±0.008	0.997 ±0.005	0.995 ±0.010	0.996 ±0.005	0.994 ±0.007	0.996 ±0.009	0.995 ±0.006	0.997 ±0.005	0.993 ±0.012	0.996 ±0.005
**precision**	0.572 ±0.048	0.656 ±0.071	0.714 ±0.037	0.962 ±0.019	0.891 ±0.046	0.952 ±0.017	0.904 ±0.029	0.997 ±0.008	0.936 ±0.020	0.999 ±0.003	0.765 ±0.063	0.998 ±0.004
**recall**	0.678 ±0.045	0.717 ±0.067	0.806 ±0.032	0.681 ±0.045	0.696 ±0.068	0.805 ±0.032	0.646 ±0.046	0.696 ±0.068	0.751 ±0.035	0.667 ±0.046	0.678 ±0.069	0.787 ±0.033
**f1**	0.615 ±0.047	0.685 ±0.069	0.757 ±0.035	0.719 ±0.044	0.782 ±0.061	0.873 ±0.027	0.669 ±0.046	0.820 ±0.057	0.833 ±0.030	0.726 ±0.043	0.719 ±0.067	0.880 ±0.026

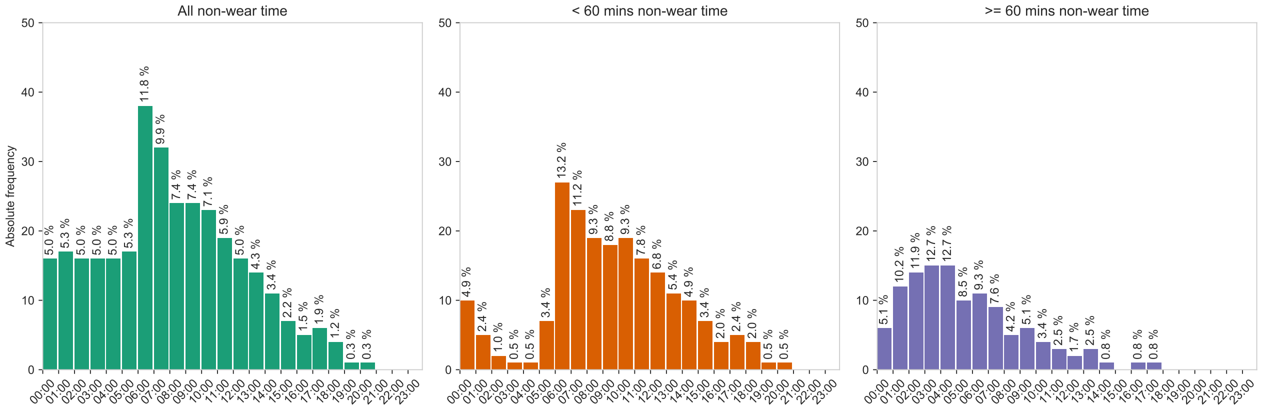

Figure S1: Distribution of non-wear time per hour of the day.

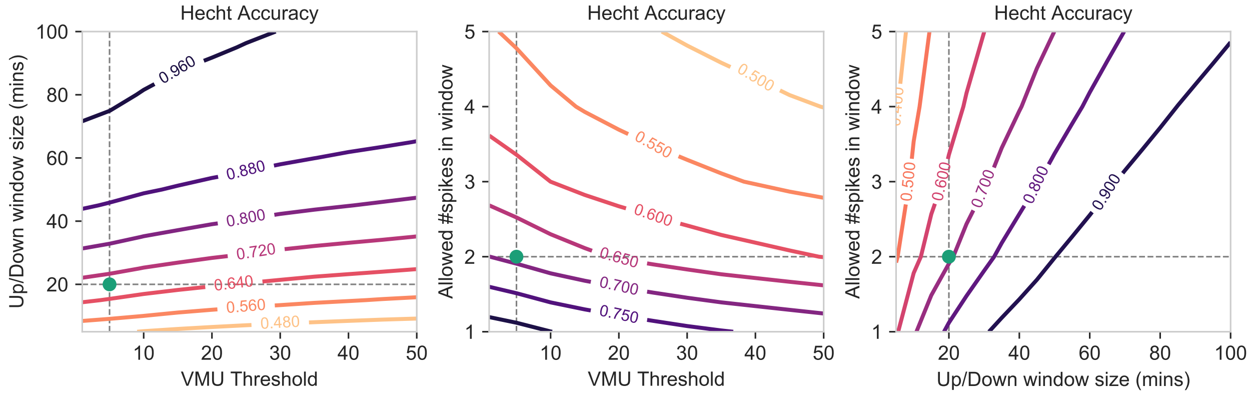

Figure S2: Contour plots showing how the accuracy score of the Hecht non-wear algorithm changes when varying the hyper-parameter values shown on the x-axis and y-axis, while keeping the remaining hyperparameters fixed to the default values. Additionally, the green dot indicates the default value of the x-axis and y-axis as originally published.

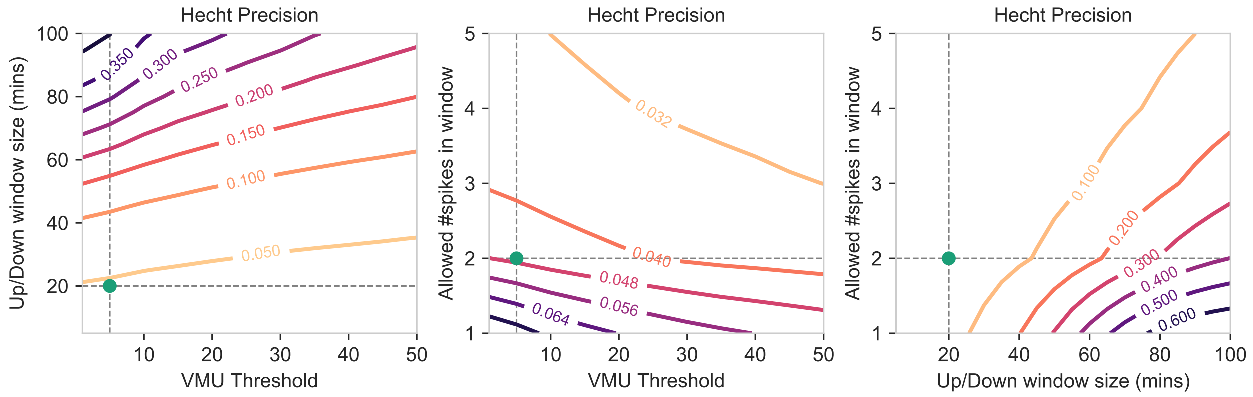

Figure S3: Contour plots showing how the precision score of the Hecht non-wear algorithm changes when varying the hyper-parameter values shown on the x-axis and y-axis, while keeping the remaining hyperparameters fixed to the default values. Additionally, the green dot indicates the default value of the x-axis and y-axis as originally published.

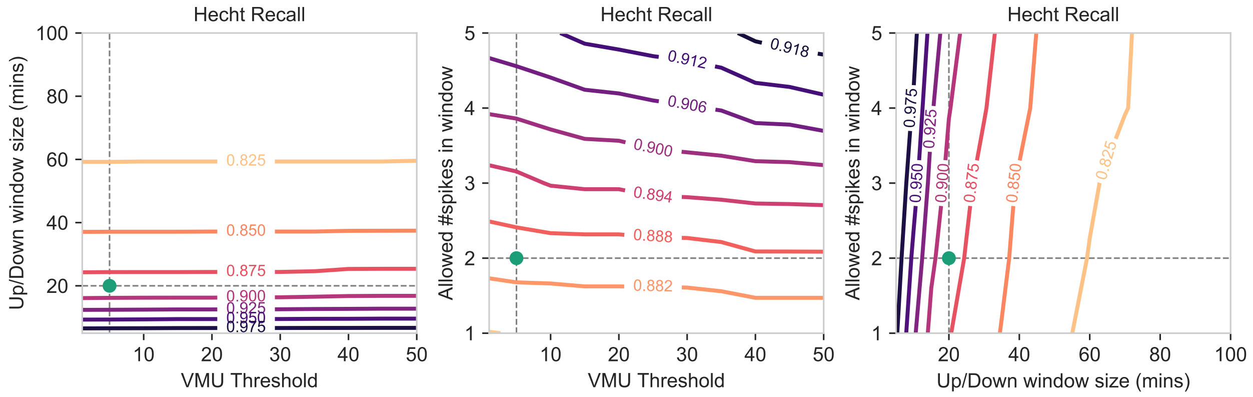

Figure S4: Contour plots showing how the recall score of the Hecht non-wear algorithm changes when varying the hyper-parameter values shown on the x-axis and y-axis, while keeping the remaining hyperparameters fixed to the default values. Additionally, the green dot indicates the default value of the x-axis and y-axis as originally published.

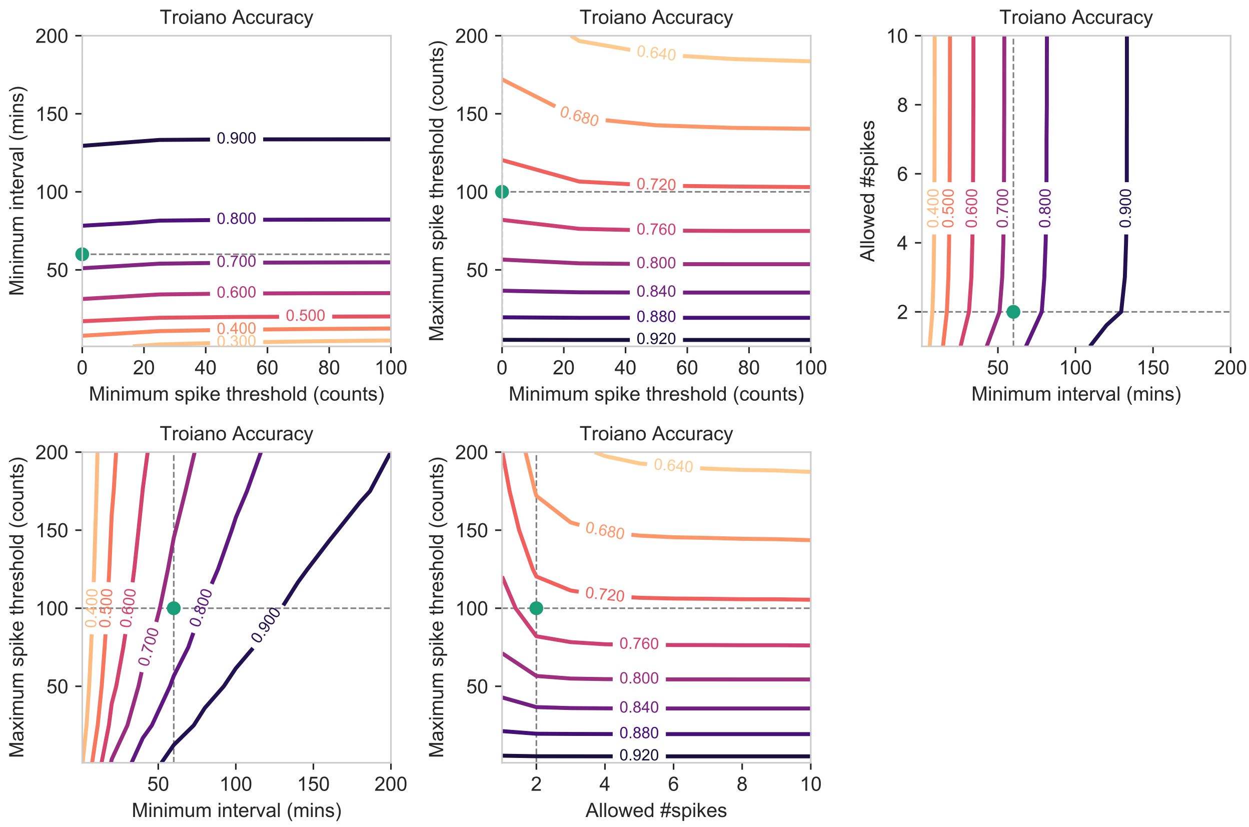

Figure S5: Contour plots showing how the accuracy score of the Troiano non-wear algorithm changes when varying the hyper-parameter values shown on the x-axis and y-axis, while keeping the remaining hyperparameters fixed to the default values. Additionally, the green dot indicates the default value of the x-axis and y-axis as originally published.

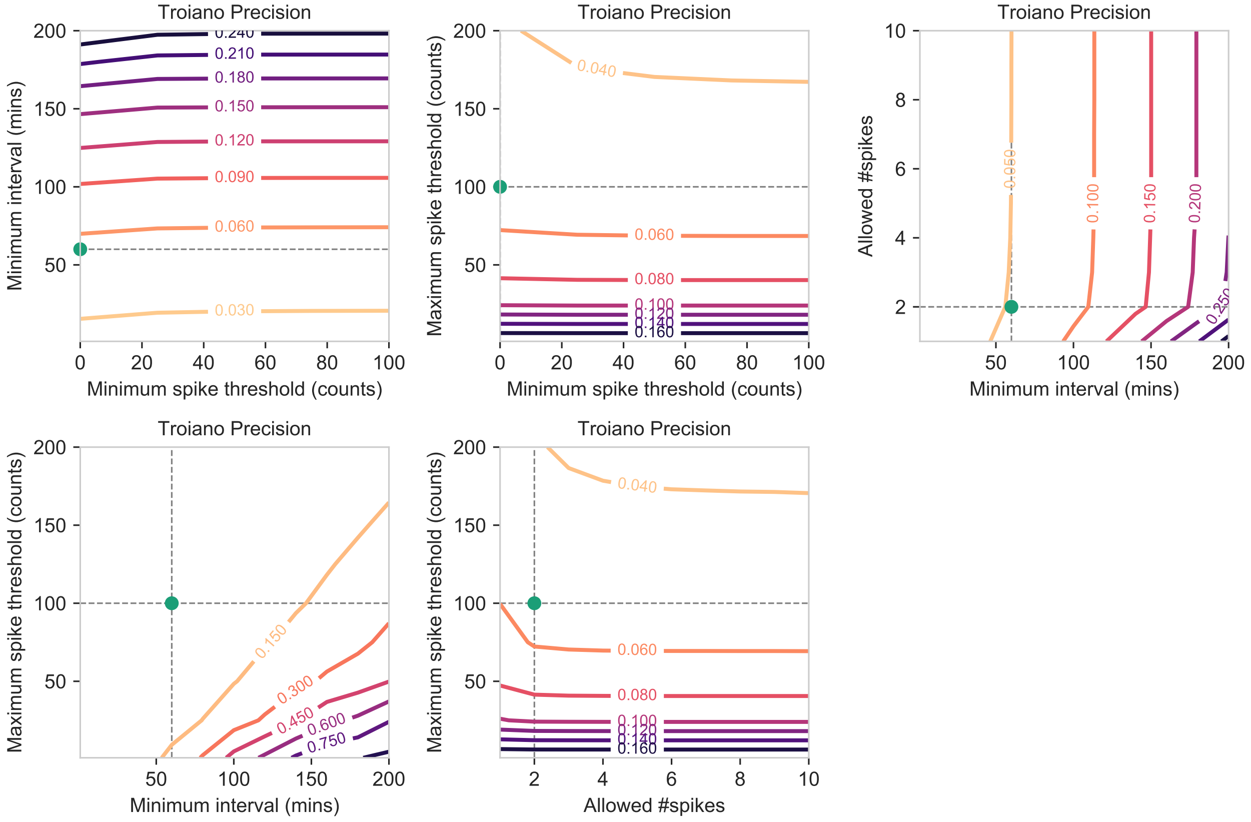

Figure S6: Contour plots showing how the precision score of the Troiano non-wear algorithm changes when varying the hyper-parameter values shown on the x-axis and y-axis, while keeping the remaining hyperparameters fixed to the default values. Additionally, the green dot indicates the default value of the x-axis and y-axis as originally published.

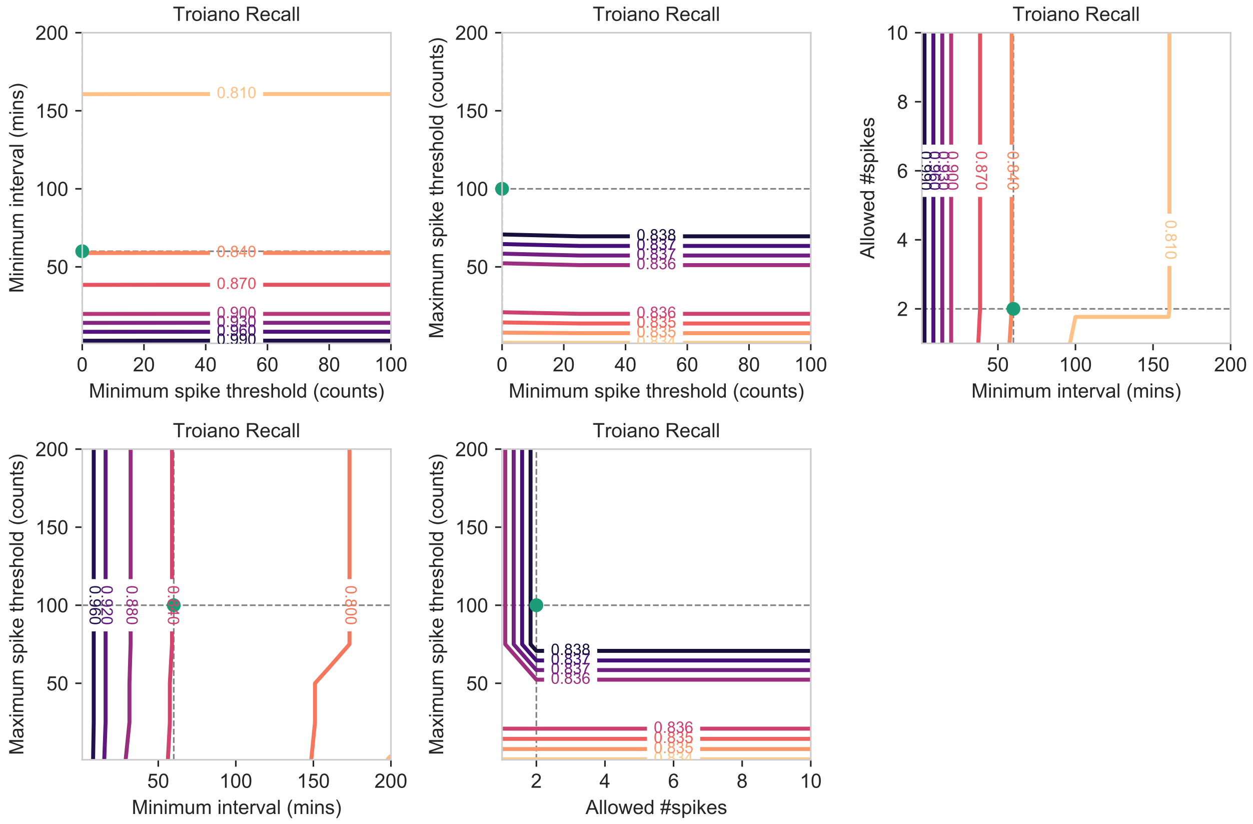

Figure S7: Contour plots showing how the recall score of the Troiano non-wear algorithm changes when varying the hyper-parameter values shown on the x-axis and y-axis, while keeping the remaining hyperparameters fixed to the default values. Additionally, the green dot indicates the default value of the x-axis and y-axis as originally published.

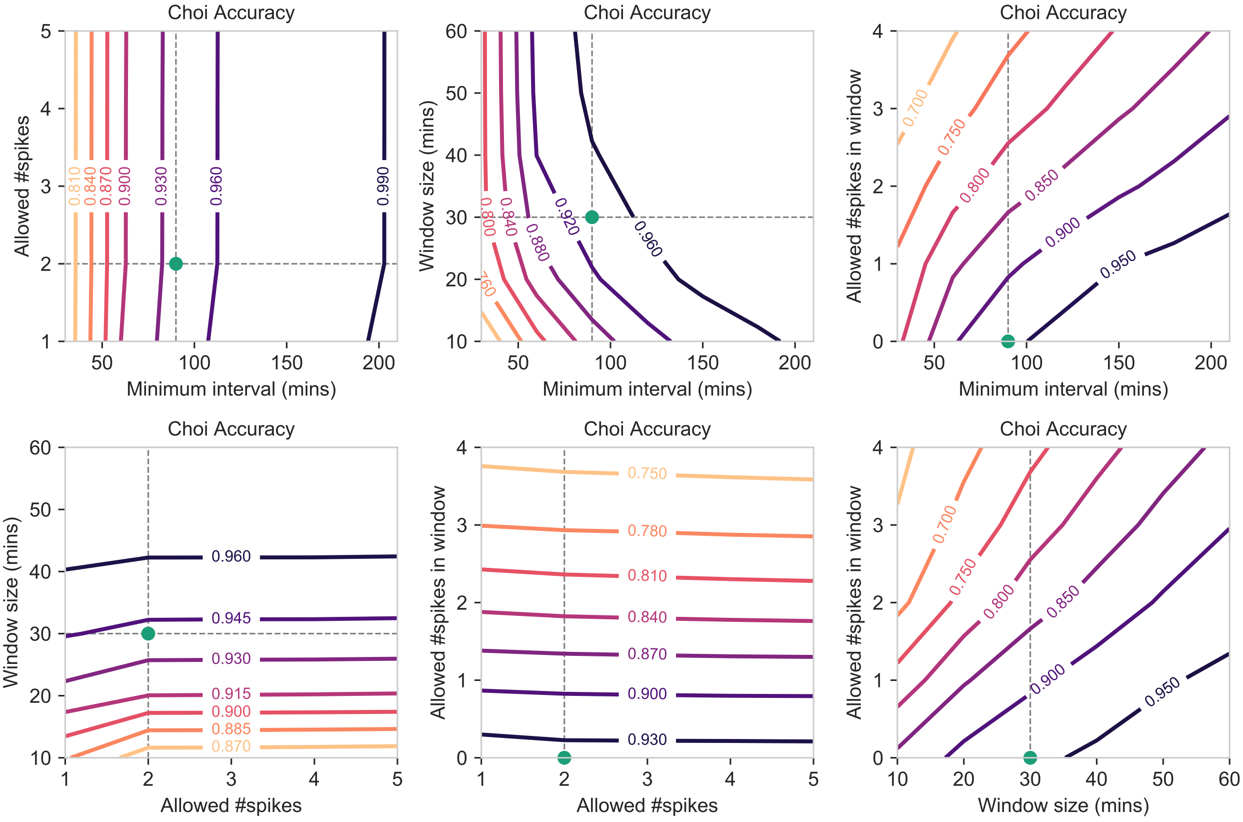

Figure S8: Contour plots showing how the accuracy score of the Choi non-wear algorithm changes when varying the hyper-parameter values shown on the x-axis and y-axis, while keeping the remaining hyperparameters fixed to the default values. Additionally, the green dot indicates the default value of the x-axis and y-axis as originally published.

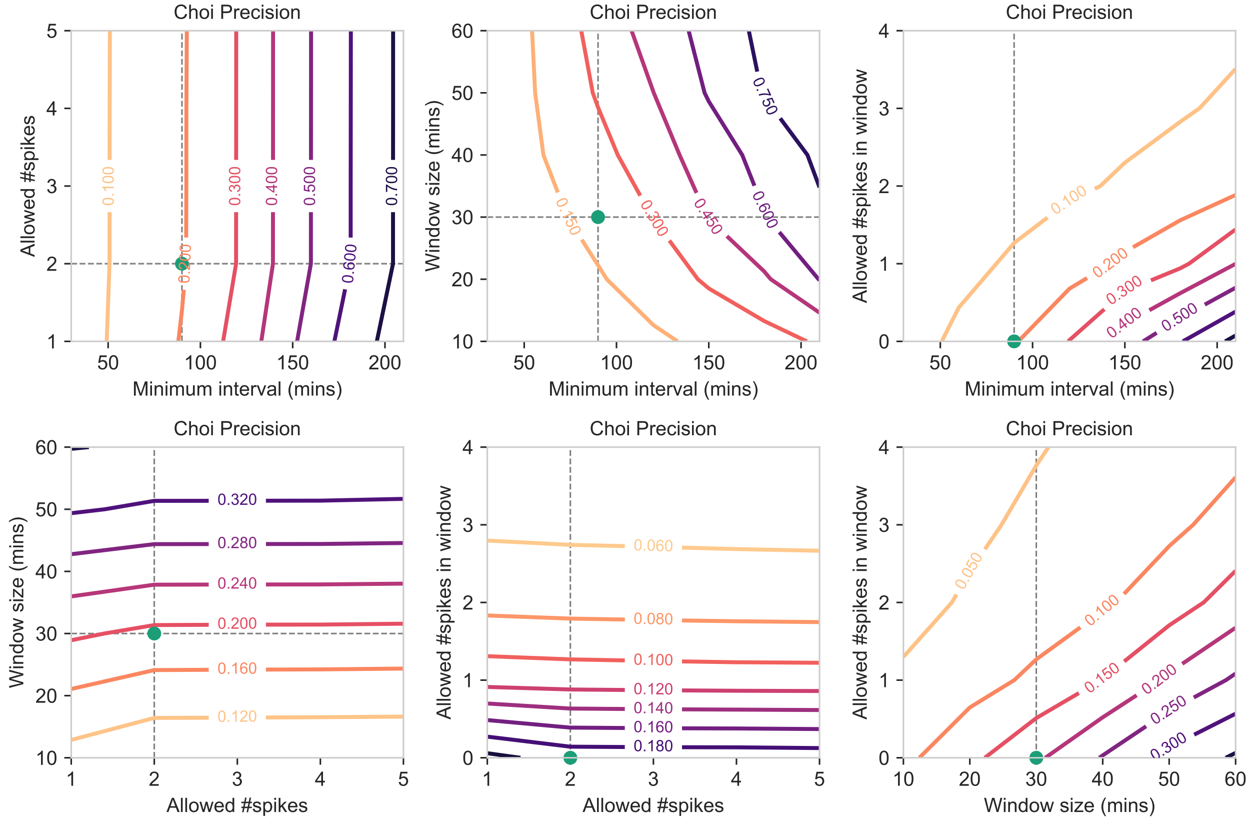

Figure S9: Contour plots showing how the precision score of the Choi non-wear algorithm changes when varying the hyper-parameter values shown on the x-axis and y-axis, while keeping the remaining hyperparameters fixed to the default values. Additionally, the green dot indicates the default value of the x-axis and y-axis as originally published.

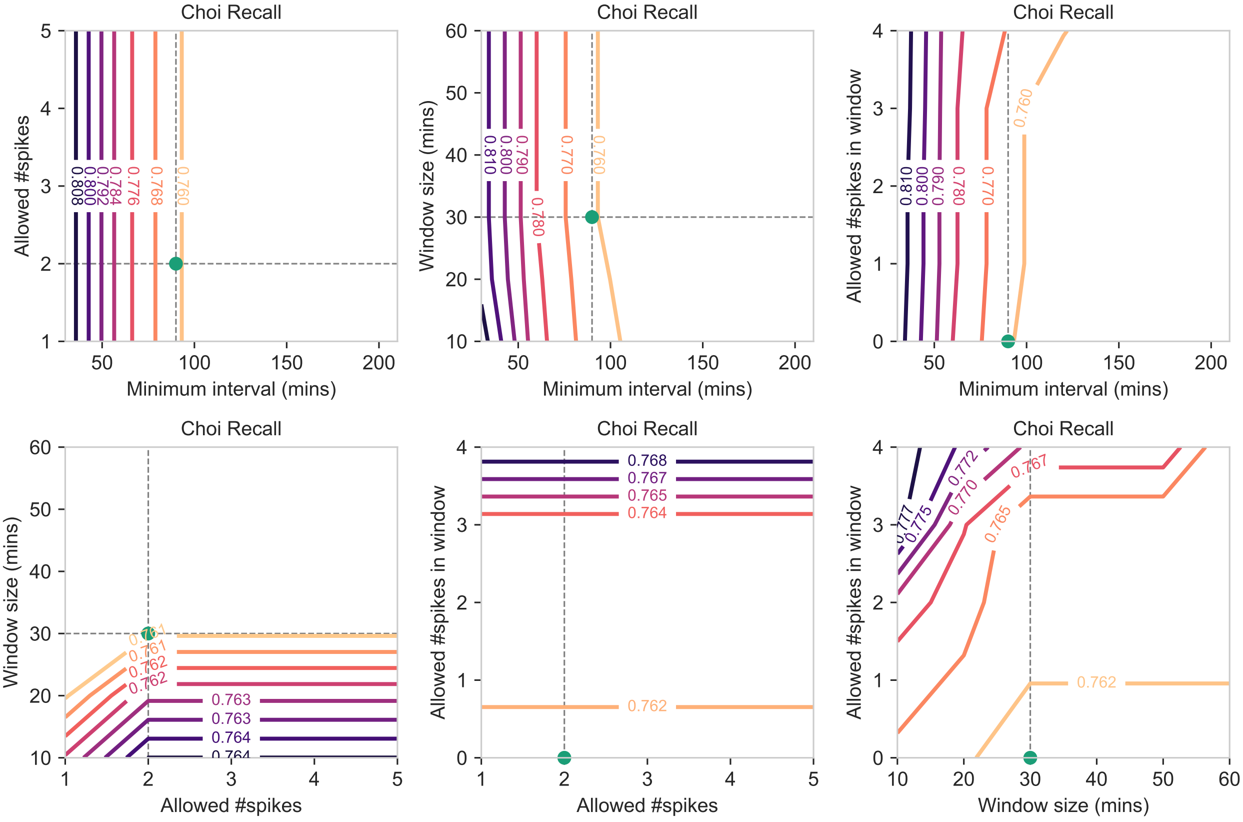

Figure S10: Contour plots showing how the recall score of the Choi non-wear algorithm changes when varying the hyper-parameter values shown on the x-axis and y-axis, while keeping the remaining hyperparameters fixed to the default values. Additionally, the green dot indicates the default value of the x-axis and y-axis as originally published.

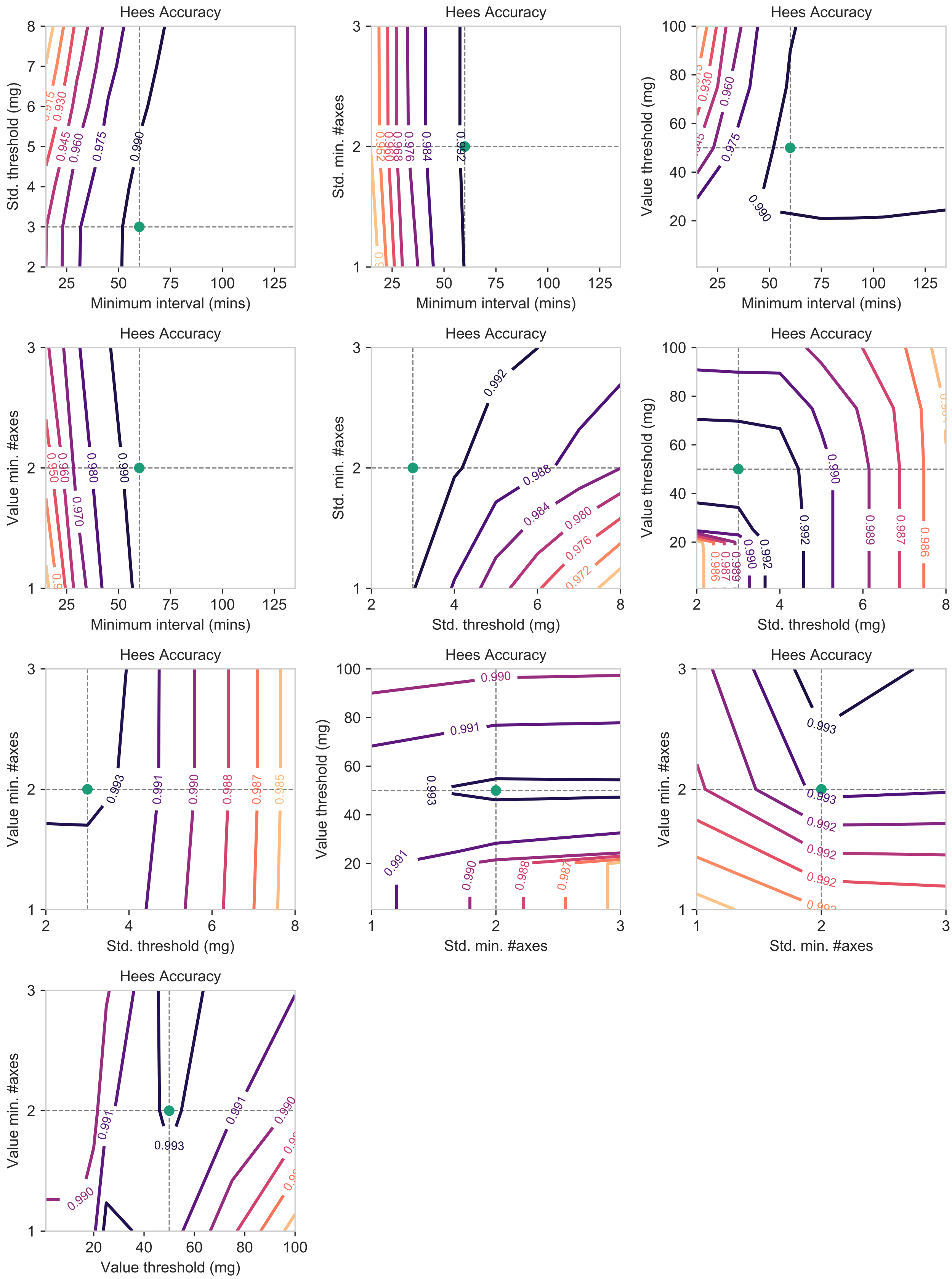

Figure S11: Contour plots showing how the accuracy score of the Hees non-wear algorithm changes when varying the hyper-parameter values shown on the x-axis and y-axis, while keeping the remaining hyperparameters fixed to the default values. Additionally, the green dot indicates the default value of the x-axis and y-axis as originally published.

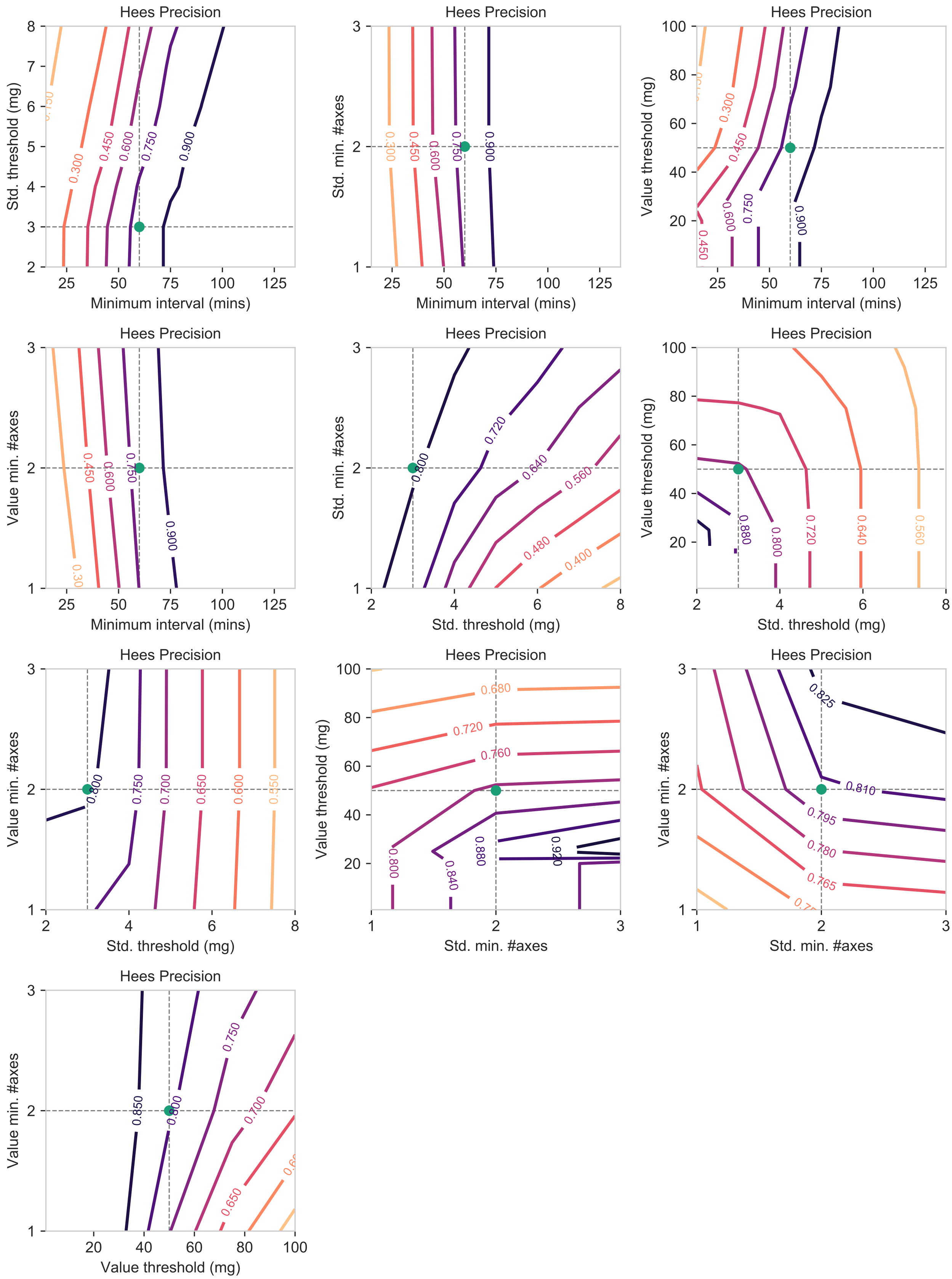

Figure S12: Contour plots showing how the precision score of the Hees non-wear algorithm changes when varying the hyper-parameter values shown on the x-axis and y-axis, while keeping the remaining hyperparameters fixed to the default values. Additionally, the green dot indicates the default value of the x-axis and y-axis as originally published.

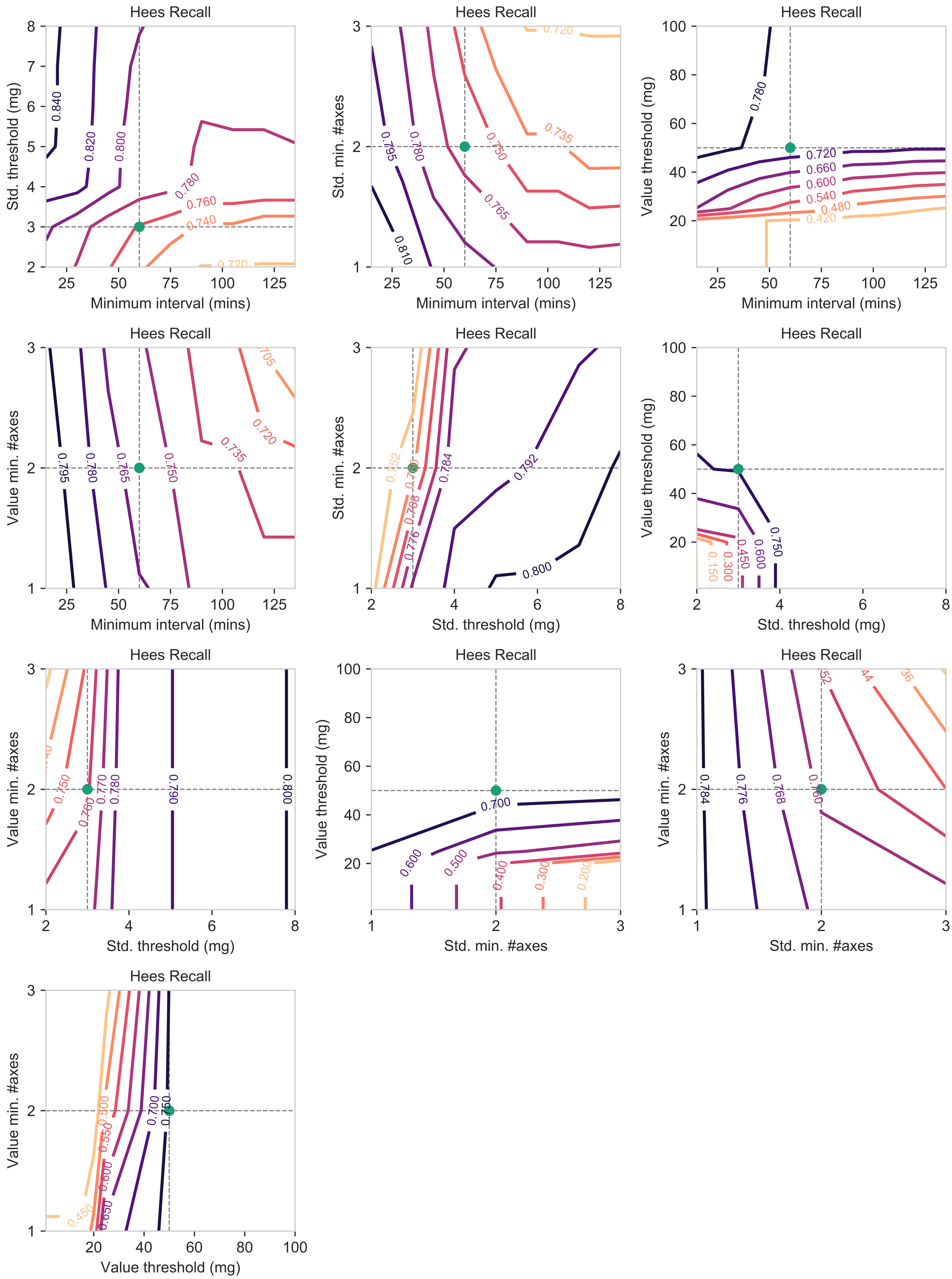

Figure S13: Contour plots showing how the recall score of the Hees non-wear algorithm changes when varying the hyper-parameter values shown on the x-axis and y-axis, while keeping the remaining hyperparameters fixed to the default values. Additionally, the green dot indicates the default value of the x-axis and y-axis as originally published.
